# Nucleosome alterations caused by mutations at modifiable histone residues in *Saccharomyces cerevisiae*

**DOI:** 10.1038/srep15583

**Published:** 2015-10-26

**Authors:** Hongde Liu, Pingyan Wang, Lingjie Liu, Zhu Min, Kun Luo, Yakun Wan

**Affiliations:** 1 State Key Laboratory of Bioelectronics, Southeast University, Nanjing 210096, China; 2 Institute of Life Sciences, Southeast University, Nanjing 210096, China; 3 Department of Neurosurgery, Xinjiang Evidence-Based Medicine Research Institute, First Affiliated Hospital of Xinjiang Medical University, Urumqi 830054, China; 4 Shanghai Institute of Materia Medica, Chinese Academy of Sciences, Shanghai 201203, China

## Abstract

Nucleosome organization exhibits dynamic properties depending on the cell state and environment. Histone proteins, fundamental components of nucleosomes, are subject to chemical modifications on particular residues. We examined the effect of substituting modifiable residues of four core histones with the non-modifiable residue alanine on nucleosome dynamics. We mapped the genome-wide nucleosomes in 22 histone mutants of *Saccharomyces cerevisiae* and compared the nucleosome alterations relative to the wild-type strain. Our results indicated that different types of histone mutation resulted in different phenotypes and a distinct reorganization of nucleosomes. Nucleosome occupancy was altered at telomeres, but not at centromeres. The first nucleosomes upstream (−1) and downstream (+1) of the transcription start site (TSS) were more dynamic than other nucleosomes. Mutations in histones affected the nucleosome array downstream of the TSS. Highly expressed genes, such as ribosome genes and genes involved in glycolysis, showed increased nucleosome occupancy in many types of histone mutant. In particular, the H3K56A mutant exhibited a high percentage of dynamic genomic regions, decreased nucleosome occupancy at telomeres, increased occupancy at the +1 and −1 nucleosomes, and a slow growth phenotype under stress conditions. Our findings provide insight into the influence of histone mutations on nucleosome dynamics.

Nucleosomes, composed of a 147-bp segment of DNA helix wrapped around a histone protein octamer, serve as the basic unit of chromatin. Studies indicate that nucleosomes play a position-specific role in transcriptional regulation by affecting DNA accessibility^1^,^2^. Typically, nucleosomes are depleted around transcription start sites (TSSs), resulting in a nucleosome-free region (NFR) that is flanked by two well-positioned nucleosomes whereas the nucleosomes downstream of the TSS are equally spaced in a nucleosome array^3^,^4^.

Nucleosome organization is highly dynamic during stem cell development^5^, upon stress^6^, and as a result of DNA mutation^7^,^8^. Histones, the basic protein components of nucleosomes, are subject to various chemical modifications, and both the N-terminal tail and the globular core of histones can be modified^9^. Histone modifications and nucleosome positioning contribute to chromatin structure^10^. The phenotypes of histone mutants have been extensively studied in *Saccharomyces cerevisiae*. The database HistoneHits currently includes the phenotypes of 677 mutants from 42 assays; the mutations include 405 of the 498 residues of yeast histones (www.histonehits.org)^11^,^12^. These analyses suggest that mutations at highly conserved residues and modifiable residues are likely to generate phenotypes^11^. Histone mutations frequently disrupt transcription and alter chromatin organization at telomeres. Dai *et al*. suggested that histone substitutions had the greatest effect on transcriptional silencing at rDNA and telomeric heterochromatic loci^12^. Triple mutation of H3K4, H3K36, and H3K79 residues to arginine is lethal and causes mitotic cell cycle delay and a progressive transcriptional defect that initiates in telomeric regions and spreads into the chromosome^13^.

Mutations of histones can also alter the genome-wide nucleosome organization^7^,^12^. With respect to the underlying mechanism, histone mutations can affect the function of chromatin remodelling enzymes, thus altering nucleosome organization. In the study by van Bakel *et al*.^7^, reductions in histone levels resulted in a shift of genic nucleosomes to positions of intrinsic sequence preference. In particular, the first downstream (+1) nucleosome relative to the TSS showed a pronounced and specific shift relative to the TSS that affected gene expression. Somers and Owen-Hughes revealed that the ATP-dependent remodelling enzymes Chd1, RSC, and SWI/SNF were affected differently by H3 N-terminal mutations, with Chd1 being the least affected and RSC being the most sensitive. Some mutations (e.g., H3I51A) prevent RSC from moving nucleosomes to locations at which the DNA is unravelled^14^. Nag *et al*. reported that the mutation H4R45C increases the accessibility of nucleosome DNA in chromatin to exogenous nucleases and may expedite nucleosomal rearrangements during nucleotide excision repair^15^.

In the present study, we determined the genome-wide nucleosome occupancy in 22 histone-mutant *S. cerevisiae* strains. The 22 histone residues can be acetylated, methylated, or phosphorylated in wild-type (WT) strains and included lysine (K), arginine (R), and serine (S) residues in the four core histones. In wild-type strains, modifications on these residues exhibit special patterns of distribution and play key roles in regulating transcription and maintaining chromatin structure^16^. We aimed to provide answers to several questions using these strains. First, do the mutations cause nucleosome reorganization? If so, what is the pattern of reorganization? Although histone deletions and substitutions are known to affect nucleosomes^7^,^15^,^17^, the genome-wide effects such as nucleosome alterations at specific genomic regions (such as the telomeres, both ends of a gene) and changes in the nucleosome array need to be explored, especially for mutations at modifiable amino acids of histones. Second, which genes are affected by histone mutations in terms of nucleosome organization? We aimed to examine the features of genes at which nucleosomes are highly reorganized in these mutants. Third, how do the histone mutants respond to stress? This analysis revealed many important details about the nucleosome dynamics of 22 histone mutants. Our results suggested that nucleosome occupancy is altered at telomeres, but not at centromeres. Moreover, the first nucleosomes upstream (−1) and downstream (+1) of TSS (the −1 and +1 nucleosomes) are highly dynamic. Highly expressed genes, such as ribosome genes and the genes involved in glycolysis, are particularly sensitive to histone mutations. Finally, different mutants show different growth phenotypes under stress conditions.

## Results

### Different histone mutations cause different alterations in nucleosome organization

This study focused on 22 mutant strains in which 22 modifiable histone residues were individually changed to alanine (Table S1 and Table S2). We determined the genome-wide nucleosome occupancy of these strains using next-generation sequencing technology.

**Figure 1:**
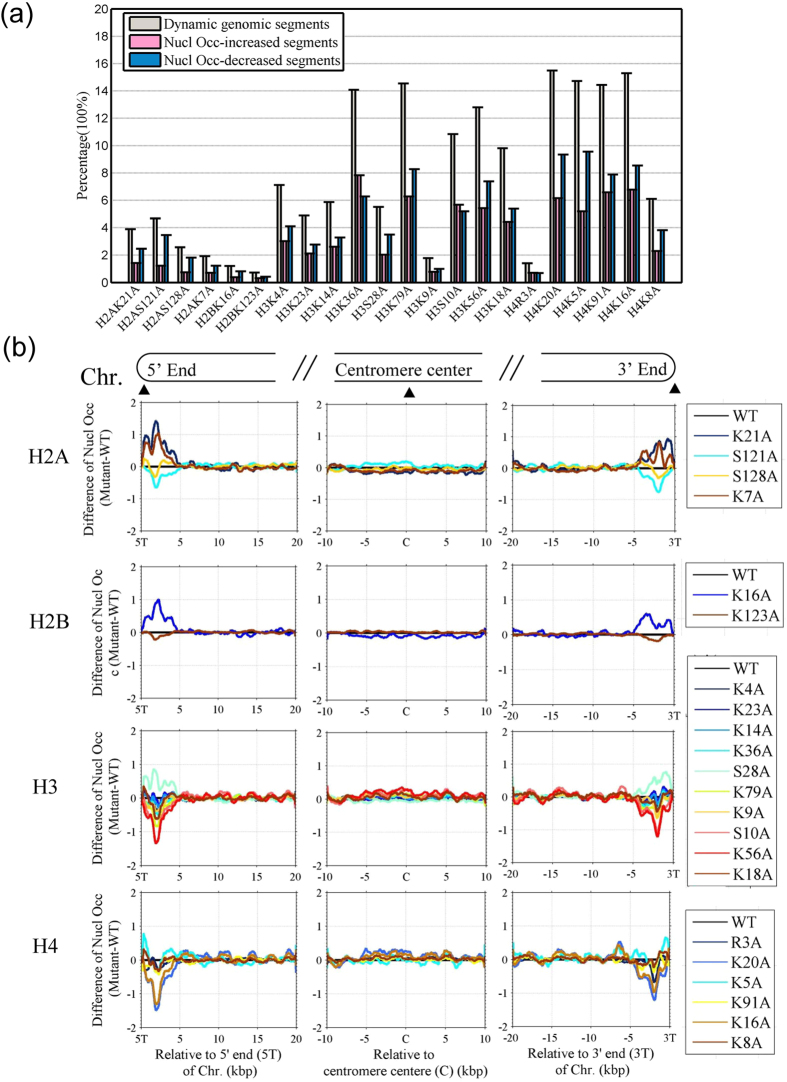
Histone mutations cause alterations in the nucleosome occupancy at telomeres. (**a**) Percentage of dynamic genomic segments in each mutant (see also Table S3). The yeast genome was divided into 24,135 continuous 0.5-kb segments. A dynamic segment was identified if the correlation coefficient (r) of nucleosome occupancy between the mutant and wild-type strain was less than 0.5 (r < 0.5) (see Methods). The genomic distribution of the dynamic segments is shown in Fig. S1a. The number of overlapping segments among the mutants is listed in Fig. S1b. (**b**) Nucleosome occupancy is greatly altered at telomeres but not at the regions around centromeres. The average difference in nucleosome occupancy between the mutant and wild-type strains at telomeres and centromeres is shown for 16 chromosomes in 22 mutants. The statistical significance of differences (P-value, two-sample *t*-test) is indicated in Fig. S3a.

In order to identify dynamic (i.e., altered) regions of nucleosome occupancy in the mutants, we divided the *S. cerevisiae* genome into 24,135 continuous 0.5-kb genomic segments and calculated the correlation coefficient of the nucleosome occupancy between the mutant and the wild-type strain for each segment. We defined a segment as ‘dynamic’ if it had a correlation coefficient less than 0.5. The 22 different histone mutations resulted in distinct nucleosome alterations (Fig. 1a and Table S3). The mutant H2BK123A had the fewest dynamic segments; only 0.72% of genomic segments were dynamic. In contrast, the mutations H4K20A, H4K16A, H4K5A, and H3K79A resulted in dynamic changes in more than 14% of segments (Fig. 1a and Table S3). At the promoter regions, the percentage of dynamic segments was lowest (5%) in mutants H2BK123A and H3K36A and highest (27%) in the H3S10A mutant (Fig. S1a). We carried out a hypergeometric test to test whether the dynamic segments are significantly located at promoters. The results showed that the segments with increased occupancy were enriched at the promoters in the mutants H3S10A, H3K18A, H4K20A, H4K5A, and H4K16A (Table S4). In contrast, the segments with decreased occupancy were not enriched at promoters in any mutant (Table S4). There was significant overlap between the dynamic segments in the following pairs of mutants: H3K9A and H4R3A, H2B123A and H2B16A, and H2B16A and H2AS128A (Fig. S1b).

We downloaded a dataset of 14 histone modifications present in wild-type yeast from the literature^16^. Using this dataset, we assessed the association between histone modification and nucleosome dynamics by calculating the correlation coefficient (Fig. S2). Our results suggest that wild-type acetylations (Ac) of H3K14, H3K18, H4K5, and H4K8 were preferentially found in nucleosome-stable segments in almost all mutants, whereas tri-methylation (Me3) at H4K20 and acetylation at H4K16 were preferentially present in nucleosome-dynamic segments. This effect was seen almost across all mutants, suggesting that histone modifications that correlate with nucleosome dynamics are independent of the mutation type, and indicating a limited combinatorial complexity of histone modifications. This is consistent with reports in the literature^16^.

### Nucleosome organization at telomeres is altered in histone mutants

Importantly, nucleosome occupancy at the telomeres exhibited substantial reorganization in the histone mutants. Nucleosome occupancy at telomeres differed from that of the wild-type strain in 15 mutants (P < 0.01, *t*-test; Fig. S3a). Compared with the wild-type strain, the occupancy was higher in mutants H2AK21A, H2AK7A, H2BK16A, H3S28A, and H4K5A and decreased in mutants H2AS121A, H3K56A, H4K20A, H4K91A, and H4K16A (Fig. 1b and Fig. S3a). This is consistent with a previous report^12^. We also noticed that the affected region was genomic region of almost 5 kb at both ends of the chromosomes, beyond the typical telomeric regions (Fig. 1b). Only three mutants, H3K18A, H2BK123A, and H2AS128A, did not show changes in nucleosomes at telomeres relative to the wild-type strain (P > 0.1) (Fig. S3a).

Although both telomeres and centromeres are associated with a heterochromatic structure, we did not observe an alteration in occupancy at centromeres of the mutant strains relative to those of the wild-type strain (Fig. 1b and Fig. S3a). Our results indicated that the chromatin at telomeres, but not at centromeres, was affected by mutations at modifiable histone residues.

Using the dataset of histone modifications present in wild-type yeast^16^, we calculated the profiles of these histone modifications for both telomeres and centromeres (Fig. S3b). There was an obvious enrichment of H3K56Ac, H4R3Me3, and H4K20Me3 at both ends of the chromosomes. Around the centromeres, H4R3Me3 was enriched whereas other modifications were depleted (Fig. S3b). This indicates that nucleosome alterations at telomeres are probably associated with the loss of some specific histone modification in the mutant strains.

### The +1 and **−**1 nucleosomes are dynamic in histone mutants

**Figure 2:**
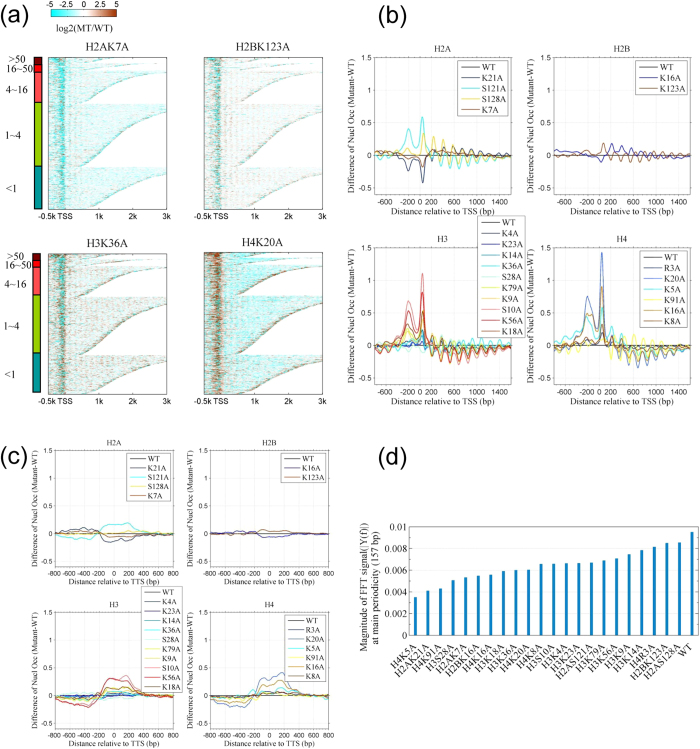
The first nucleosomes upstream (−1) and downstream (+1) of a transcription start site (TSS) are dynamic in histone mutants. (**a**) Heat maps showing the log_2_ fold changes in nucleosome occupancy in the histone mutant strains relative to the wild-type strain for 5,419 genes. Data were aligned according to the TSS and grouped by transcription rate (rate < 1, 1 to <4, 4 to <16, 16 to <50, and ≥ 50; within each group, the genes were sorted by length). Data from mutants H2AK7A, H2BK123A, H3K36A, and H4K20A are shown. Nucleosome occupancy profiles are shown in Fig. S5a. (**b**) Differences in nucleosome occupancy around the TSS **b**etween mutant and wild-type strains. Each profile represents the average difference in occupancy between the mutant and the wild-type strain for 5,419 genes. The variation of dyad position and occupancy is shown in Fig. S4a. The significance of differences in occupancy is given in Fig. S4b. (**c**) Differences in nucleosome occupancy around the trans**c**ription termination sites between mutant and wild-type strains (analysis as for Fig. 2b; see also Fig. S4b). (**d**) Histone mutations disrupt the periodicity of the nucleosome distribution. The bar plot shows the intensities of Fourier spectra (see Fig. S6) of the distributions of nucleosomes with a periodicity of 157 bp, where the Fourier spectra highly peak, in the 22 mutants and the wild-type strains. The periodicity was calculated using the autocorrelation signal of the nucleosome occupancy profile of 16 chromosomes.

The +1 and −1 nucleosomes tend to be dynamically regulated according to cell-associated changes^18^,^19^. Here, we revealed that the +1 and −1 nucleosomes exhibited large occupancy changes in all 22 histone mutants (Fig. 2). We plotted the fold change (log_2_) of nucleosome occupancy of 5,419 genes between the mutant and the wild-type strains (Fig. 2a, only four mutants are shown because of limited space). The results indicate the obvious dynamics of nucleosome occupancy at gene promoters (Fig. 2a). We then plotted the average difference in nucleosome occupancy of the 5,419 genes between the mutant and wild-type strains (Fig. 2b). Consistent with Fig. 2a, b shows a high peak or a low valley at the +1 and −1 nucleosome positions. In mutants of histones H3 and H4, occupancy of the +1 and −1 nucleosomes was drastically increased (Fig. 2b and Fig. S4a [right]), whereas the occupancy of these two nucleosomes was decreased in mutants H2AK21A and H2AK7A (Fig. 2b). A large shift in dyad position was observed at the −1 nucleosome between the mutant and the wild-type strains (Fig. S4a [left]), suggesting a fuzzy positioning at the −1 nucleosome. In contrast, the +1 nucleosomal dyad position showed a small shift but a great occupancy alteration (Fig. S4a). In a further analysis (Fig. S4b [left]), a difference in occupancy was observed at the +1 nucleosome in mutants H2AS121A, H3S10A, and H4K20A (P < 10^−20^, *t*-test) and a difference at the +2 nucleosome was found in mutant H2BK16A. The nucleosome profiles and detailed dynamics of the +1 and −1 nucleosomes are shown in Fig. S5.

The nucleosome occupancy was also altered near transcription termination sites (TTSs), although the change in occupancy was less than 0.5-fold in all of the mutants (Fig. 2c). Within a 400-bp vicinity of TTSs, the average nucleosome occupancy showed a change in mutants H2AS121A, H3S10A, and H4K20A compared with the wild-type (P < 10^−10^, *t*-test) (Fig. S4b [right]).

A distinctive feature of nucleosome distribution is that nucleosomes downstream of TSSs are phased, forming a nucleosome array^2^. Thus, the nucleosome distribution shows a periodicity determined by the length of core DNA (147 bp) and linker DNA. To test whether histone mutations disrupt the nucleosome array, we examined the magnitude of the periodicity in mutant and wild-type strains (Fig. 2d and Fig. S6). As expected, the magnitude of periodicity was lower in the mutants than in the wild-type strain, suggesting that histone mutations alter the phased nucleosome distribution downstream of TSSs.

### Nucleosome occupancy at the promoters of highly expressed genes is greatly affected in histone mutants

**Figure 3:**
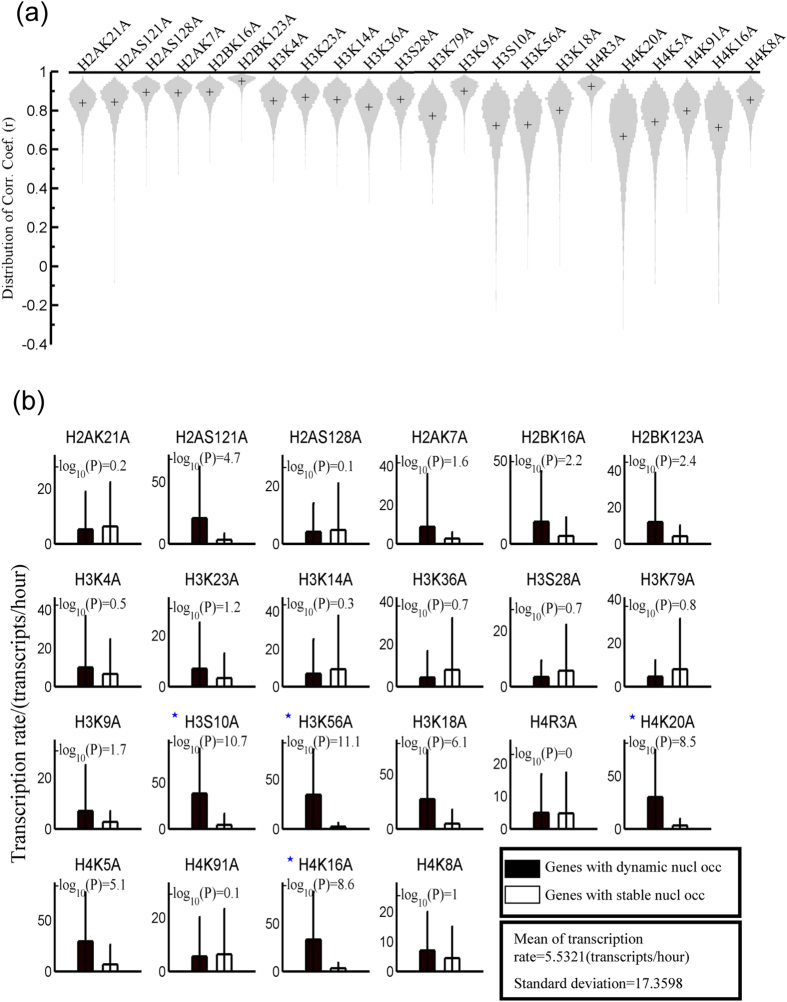
Highly expressed genes show a greater alteration in nucleosome occupancy at promoters upon histone mutation than poorly expressed genes. (**a**) Violin plot showing distributions of correlation coefficients (r) between the mutant and wild-type strains at the promoter regions (−0.5 kb to 0.3 kb) of 5,419 genes; “+” indicates the mean of the coefficients. Lower coefficients indicate more dynamic nucleosome occupancies. (**b**) Transcription rate of the top 108 dynamic genes and top 108 stable genes in the wild-type strain. First, the correlation coefficients were sorted, and then the top 108 genes with high coefficients were chosen as stable genes, and the top 108 genes with low coefficients were chosen as dynamic genes. Finally, the transcription rates were compared between the dynamic and stable genes using a two-sample *t*-test. Data on transcription rates for wild-type strains were retrieved from the literature^54^. The statistical significance of differences is indicated (–log_10_ P-values); asterisks (*) indicate the mutants with P-values < 10^−8^.

As shown in Fig. 2b, nucleosomes near TSSs show dynamic properties upon histone mutation. We further identified genes with altered occupancy at their promoter by calculating the correlation coefficient of the nucleosome occupancy profiles of the mutant and wild-type strains; a low coefficient, <0.5, indicates that the nucleosome occupancy is significantly altered. The distribution of the coefficients of 5,419 genes for each mutant is presented as a violin plot in Fig. 3a. In five mutants (H3S10A, H3K56A, H4K20A, H4K5A, and H4K16A), many of the genes had a low correlation coefficient, indicating that these five histone mutations affected the nucleosome occupancy of a greater number of genes than other mutations (Fig. 3a). The mutants H2BK123A, H3R4A, and H3K9A had a smaller effect on the nucleosome occupancy at promoters. Bi-clustering analysis of the correlations of 5,419 genes in 22 mutants suggested that different mutations have distinct effects on nucleosome occupancy (Fig. S7).

We selected the 108 (2% of 5,419 genes) most dynamic genes and the 108 most stable genes, which were associated with the lowest and the highest correlation coefficients, respectively. We compared the gene transcription rates of the two types of genes in the wild-type strain. Dynamic genes showed a higher transcription rate than the stable genes, especially in mutants H3S10A, H3K56A, H4K20A, H4K5A, and H4K16A (Fig. 3b). Histone modifications showed a non-regular profile in the dynamic genes in wild-type strains (Fig. S8). This suggests that nucleosomes of these highly expressed genes are reorganized in strains with histone mutations and that the nucleosome occupancy of poorly expressed genes will be less affected by histone mutations.

### Ribosome genes and genes associated with glycolysis are common dynamic genes with an increased nucleosome occupancy in histone mutants

**Figure 4:**
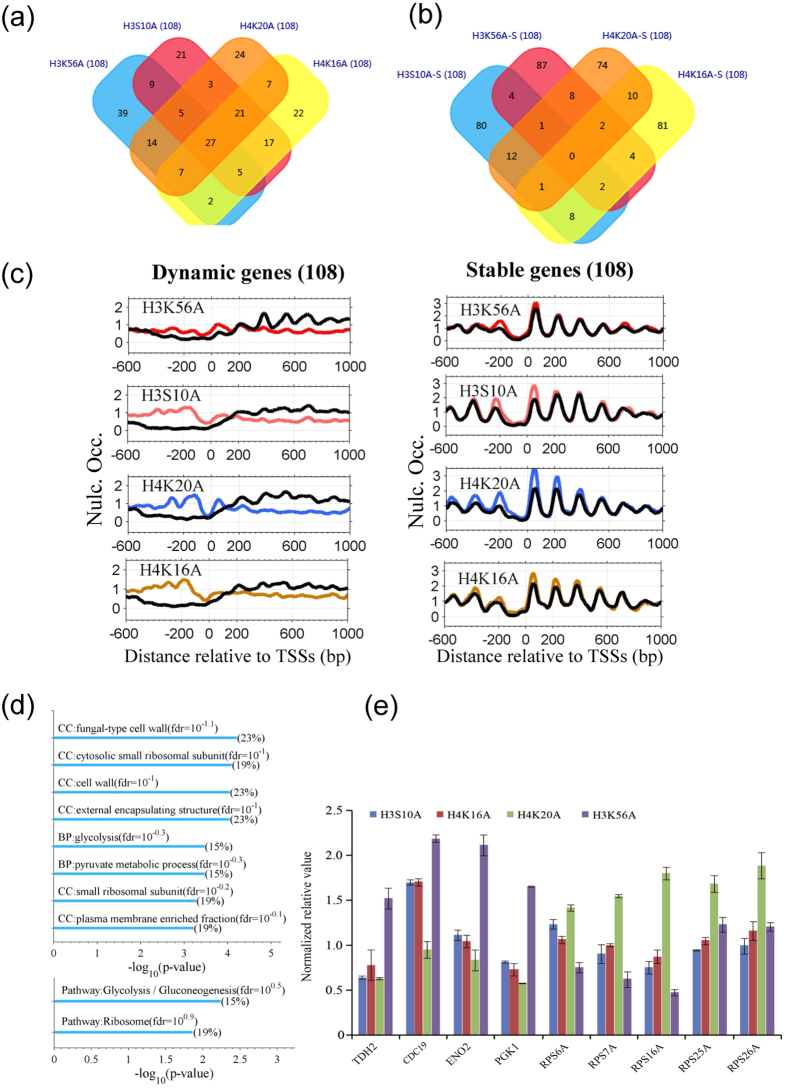
Dynamic genes show a high degree of overlap among mutants H3S10A, H3K56A, H4K16A, and H4K20A, and are significantly enriched in glycolysis (gluconeogenesis) and ribosome pathways. (**a**) Venn diagram showing the number of overlapping dynamic genes in the H3S10A, H3K56A, H4K16A, and H4K20A mutants. Additional information is provided in Fig. S9. (**b**) Same as (**a**) except for overlapping stable genes. (**c**) Nucleosome occupancy profiles around TSSs of the 108 dynamic genes (left panels) and 108 stable genes (right panels). (**d**) Enrichment analysis of the Kyoto Encyclopedia of Genes and Genomes pathways and Gene Ontology (GO) for 27 common dynamic genes in mutants H3S10A, H3K56A, H4K16A, and H4K20A. BP: biological processes; CC: cellular components. Additional GO enrichment analysis for the dynamic genes is shown in Fig. S10. (**e**) Expression changes in common dynamic genes (*TDH2*, *CDC19*, *ENO2*, *PGK1*, *RPS6A*, *RPS7A*, *RPS16A*, and *RPS26A*) in the mutant (H3S10A, H3K56A, H4K16A, and H4K20A) and wild-type strains were examined by quantitative reverse transcription polymerase chain reaction (RT-PCR). The experiments were repeated three times for each gene in each mutant. The nucleosome occupancy profiles for *TDH2*, *CDC19*, and *RPS16A* are shown in Fig. S9c–e.

We selected the top 108 dynamic genes in each mutant and determined whether there was an overlap in dynamic genes among the mutants. A Venn diagram indicates the overlapping genes in the mutants H3S10A, H3K56A, H4K20A, and H4K16A (Fig. 4a and Fig. S9a). Twenty-seven genes were found to be dynamic in all four mutant strains (P = 1.9 × 10^−190^, hypergeometric distribution) (Fig. 4a). Importantly, no common stable genes among the four mutants were identified (Fig. 4b). This indicated that alteration in nucleosome occupancy due to histone mutations frequently occur in the same genes.

We next examined the nucleosome occupancy profile for the top 108 dynamic genes. Consistent with their high level of expression (Fig. 3b), in the wild-type strain the dynamic genes had a 0.5-kb nucleosome-depleted region at their promoter (Fig. 4c left, black lines). Upon substitution at some modifiable histone residues, the nucleosome occupancy consistently increased at the promoters and decreased in the gene bodies (Fig. 4c left). The nucleosome occupancy profiles for three of the common dynamic genes are shown in Fig. S9c–e, and the results are consistent with those shown in Fig. 4c.

We then carried out an enrichment analysis for the 27 common dynamic genes using Gene Ontology (GO) terms and Kyoto Encyclopedia of Genes and Genomes (KEGG) pathways (Fig. 4d and Fig. S10). The common dynamic genes were enriched in pathways of both ribosome and glycolysis and GO terms (cellular components) associated with cell wall and plasma membrane (Fig. 4d). Because ribosome- and glycolysis-associated genes are related to mRNA translation and energy generation, we inferred that histone mutations affect both protein production and metabolism. Repression of ribosome genes under stress conditions has previously been suggested^20^. From this perspective, a histone mutation would probably represent a type of cellular stresses.

We also determined expression changes for 9 of the 27 common dynamic genes by RT-PCR. Working on the principle that increased nucleosome occupancy at the promoter, especially at a nucleosome-depleted region, will inhibit transcription to some degree^21^, the genes with increased nucleosome occupancy were expected to have lower expression in the mutants than in the wild-type strain. Unexpectedly, we did not observe a consistent decrease in gene expression (fold change <1) in the mutants by RT-PCR (Fig. 4e).

It should be noted that western blot analysis for H3 protein in wild-type and mutant strains confirmed that there was no difference in the expression levels of histone H3 between the wild-type strain and the histone mutant strains (Fig. S11).

### Growth of mutants under stress condition**s**

**Figure 5:**
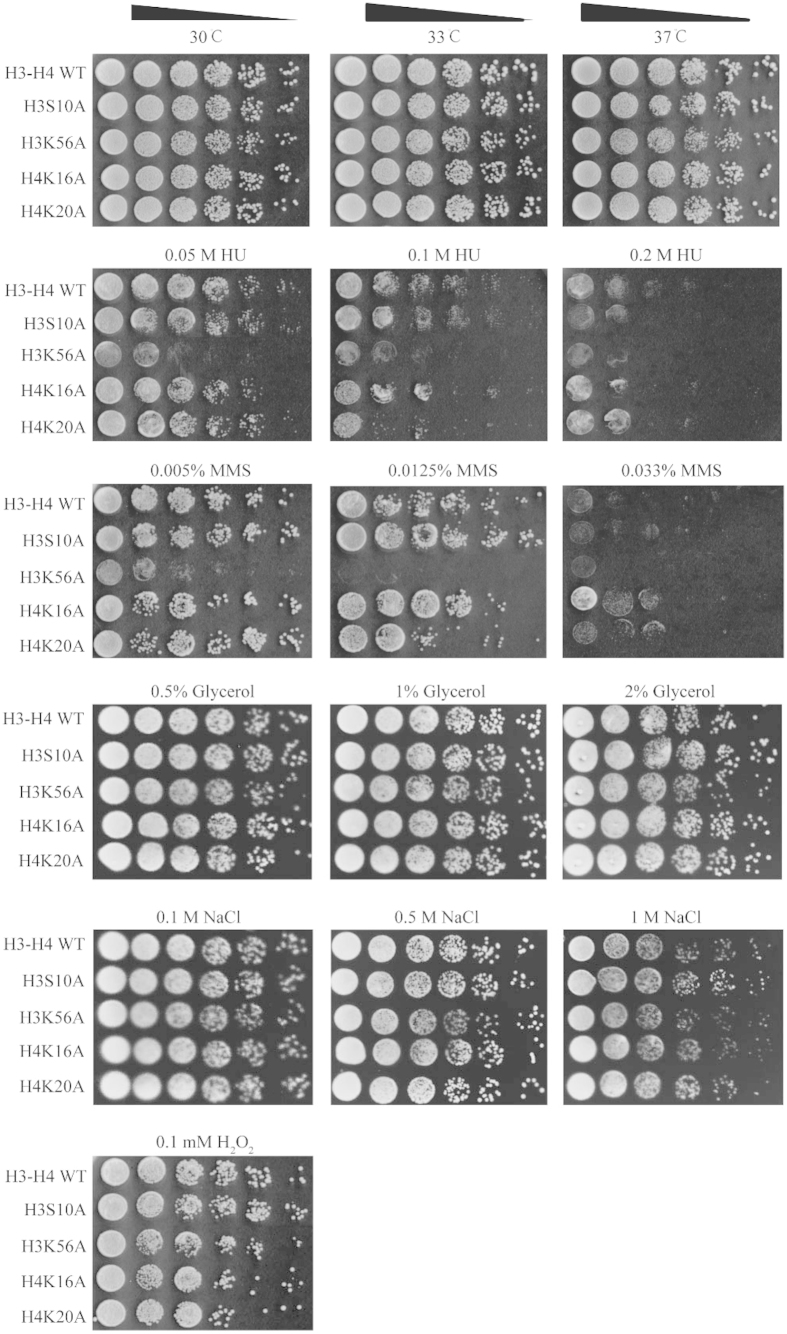
Growth of the mutants under different stress conditions. In spot assays, mutants H3S10A, H3K56A, H4K16A, and H4K20A were grown with 0.05 M hydroxyurea (HU), 0.1 M HU, 0.2 M HU, 0.005% methyl methanesulfonate (MMS), 0.0125% MMS, 0.033% MMS, 0.5% glycerol, 1% glycerol, 2% glycerol, 0.1 M NaCl, 0.5 M NaCl, 1 M NaCl, or 0.1 mM H_2_O_2_. Additionally, growth in yeast extract, peptone, and dextrose medium at 30 °C, 33 °C, and 37 °C was examined to determine temperature sensitivity.

Finally, we performed a spot assay to measure growth of the mutants H3S10A, H3K56A, H4K16A, and H4K20A under various stress conditions. The mutant H3K56A exhibited repressed growth on both methyl methanesulfonate (MMS) and hydroxyurea (HU) compared with the wild-type strain (Fig. 5). This is probably due to the lack of H3K56 acetylation that causes checkpoint deactivation^22^,^23^. The data represented in Fig. 5 also suggest that growth of the four mutants was decreased upon alteration of the carbon source from glucose to glycerol. Combined with the finding that nucleosome dynamics of glycolysis-associated genes were affected (Fig. 4d), we speculate that these histone mutations might affect carbon usage, resulting in a growth defect.

## Discussion

Chromatin organization is governed by multiple factors, such as DNA sequence, the chromatin-remodelling complex, histone modifications, histone variants, transcription factor binding, and the interactions among them^1^,^4^,^24^,^25^. Substituting modifiable residues with alanine directly results in a loss of histone acetylation and methylation^26^ and alters the function of the chromatin-remodelling complex^9^,^14^. Through these processes, nucleosome organization is affected. Here, we revealed nucleosome occupancy alterations in 22 mutants in which the modifiable lysine, serine, and arginine residues were individually changed to alanine. In particular, we observed alterations in nucleosome occupancy at both telomeres and promoters.

**Figure 6:**
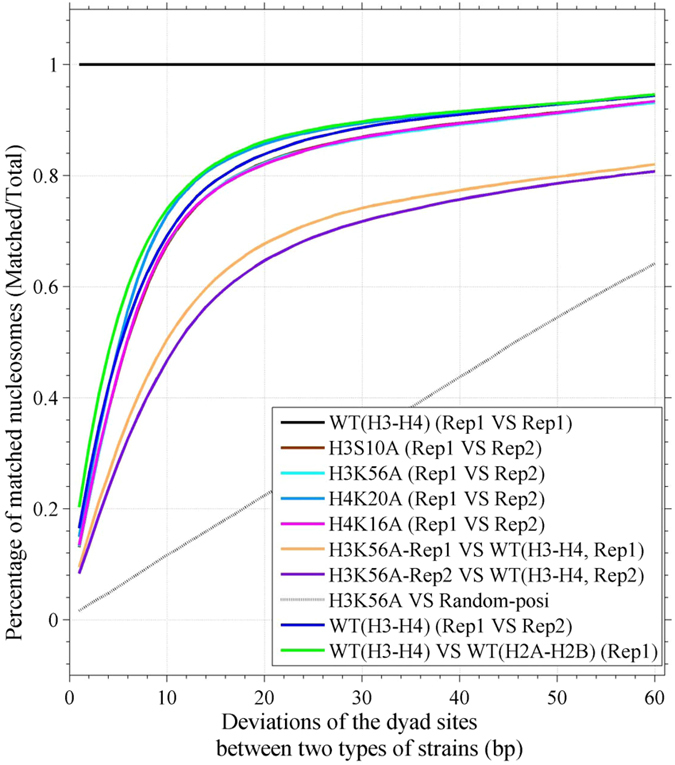
Repeatability test between two biologic replications assessing nucleosome matching percentage under a varying deviation. Nucleosome matching percentage is the percentage value of the number of matched nucleosomes between two replications (Rep1 and Rep2) relative to the total number of identified nucleosomes in the first replication (Rep1) under a certain deviation. The deviation varied from 1 bp to 60 bp. The dyad positions of nucleosomes were identified with a wavelet transformation-based peak finding algorithm. Nucleosome matching percentage between two biologic replications was calculated for H3S10A, H3K56A, H4K20A, and H4K16A mutant strains and for H3 and H4 wild-type (WT) strains, respectively. The matching percentage between the WT and mutant H3K56A is also shown. The green line shows the average matching percentage between the WT (WT H2A-H2B versus WT H3-H4) in the first experiment (Rep1). The blue line indicates the matching percentage between two replications of WT H3-H4.

To test whether these alterations were due to experimental fluctuations, we carried out a biologic replication test for mutants H3S10A, H3K56A, H4K20A, H4K16A and wild-type strains. We calculated the nucleosome matching percentage between two types of strains at a given deviation, which ranged from 1 bp to 60 bp (Fig. 6). Given a deviation, the matching percentage is the percentage value for the number of matched nucleosomes lower than the deviation between two types of strains relative to the total number of identified nucleosomes in the wild-type strain (or replication 1). We observed a high matching percentage between two replications (Rep1 and Rep2) for either mutant or wild-type strains, indicating a good repeatability. Importantly, the matching percentages between two wild types and between two replications of the mutant were much higher than those between the mutant and the wild type, suggesting that the nucleosome alteration observed between the mutant and the wild-type strains cannot be attributed to experimental fluctuations, but is due to the mutations in the histones. We also repeated the nucleosome profiling for any genomic region and the telomeres (Fig. S12) using the dataset of the second biologic experiment.

Our results raise four important points. First, different types of histone mutations lead to different phenotypes and distinct reorganization of the nucleosomes. In this study, two mutants of H2B showed a slight nucleosome alteration (Figs 1 and 2). In contrast, mutants of histones H3 and H4 showed a great change in nucleosome organization at both telomeres and promoters.

Second, both telomeres and promoters undergo a great change in nucleosome occupancy in histone mutant strains (Figs 1 and 2). Heterochromatin of telomeres is closely associated with trimethylation histone modifications of H3K9 (H3K9me3), H3K79me3, and H4K20me3^27^,^28^. Loss of these modifications is known to affect telomere structure. Here, we revealed that, in addition to H4K20A, H3K9A, and H3K79A, mutations in H4K91, H3K56, H2BK16A, and other mutations can affect nucleosome occupancy at telomeres (Fig. 1 and Fig. S3a), suggesting that several histone modifications are associated with telomere heterochromatin. In wild-type yeast, acetylation at H3K56 and methylation at both H4R3 and H4K20 is enhanced at the telomeres (Fig. S3b). Conversely, in regions around the centromeres histone modifications are depleted, with the exception of H4R3 methylation. This suggests an intimate association between nucleosome dynamics and the loss of histone modification in the mutant strains.

Nucleosomes at promoters are involved in transcriptional regulation. Nucleosomes at promoter regions, especially the +1 and −1 nucleosomes, are enriched for both histone modifications and the histone variant H2A.Z^29^,^30^ and are also a target region of ATP-dependent remodelling enzymes and polymerase^14^,^31^,^32^. In the histone mutants, the dyad position of the −1 nucleosome is greatly altered, whereas that of the +1 nucleosome is less affected (Fig. S4). The +1 nucleosome is vital for forming the nucleosome array downstream of TSSs, initiating transcription^33^,^34^, and regulating elongation^35^. Thus, we inferred that variation of the dyad position of the +1 nucleosome is restricted to a limited range, even upon histone mutation. Pugh *et al*. suggested that the −1 nucleosome would be removed upon recruitment of Pol II during transcription^36^. If a histone mutation alters transcription^12^, the dyad position of the −1 nucleosome will be shifted or removed, resulting in a great variation of the dyad position. We speculate that the promoter nucleosome dynamics are coupled with the loss of histone modifications and transcriptional alterations in the histone mutants.

Third, histone mutations affect the nucleosome occupancy of genes with high expression. We revealed that nucleosome occupancy is increased on both ribosome genes and genes involved in glycolysis in histone mutants (Figs 3 and 4). However, unlike a previous report^12^, we did not observe a consistent decrease in the expression of genes with increased nucleosome occupancy (Fig. 4e). This discrepancy is probably because nucleosome occupancy at promoters is not directly correlated with the transcription level^37^,^38^,^39^,^40^ and because the transcription level is probably governed by multiple factors through complicated mechanisms.

Fourth, the H3K56A mutant exhibits particular characteristics including a high percentage of dynamic genomic regions, decreased nucleosome occupancy at telomeres (Fig. 1), increased occupancy at the +1 and −1 nucleosomes (Fig. 2), and slow growth under stresses (Fig. 5). Acetylation of H3K56, which is accomplished by the acetyltransferase Rtt109 and recognized by chaperones Rtt106, CAF1, and Asf1, is implicated in DNA replication and repair, nucleosome disassembly during transcription, telomere peripheral positioning, and chromosome folding^41^,^42^,^43^,^44^,^45^. The acetylation/deacetylation cycle of the H3K56 residue was found to be required for proper telomere localization^44^. Kaplan *et al*. suggested that Rtt109 is active at rapidly replaced nucleosomes and that H3K56 acetylation enhances subsequent replacement at the same location^46^. This likely explains the alterations in nucleosomes at promoters in the mutant H3K56A strain in our study. Also, reports in the literature suggest that restoration of the chromatin following double-strand break (DSB) repair is driven by H3K56 acetylation and that this is a signal for completion of repair. Thus, the lack of H3K56 acetylation probably causes checkpoint deactivation^22^,^23^. This might be related to the slow growth of the H3K56A mutant under conditions of stress.

Finally, we also observed several features of nucleosome dynamics that were similar among the mutants. First, promoter regions are not enriched in the occupancy-decreased segments (Table S4), meaning that nucleosome occupancy is not decreased at most of the promoters in most of the mutants (Fig. 2b). Given that nucleosome occupancy near TSSs blocks the accessibility of DNA for transcription^21^,^38^,^40^,^47^, our result likely suggests no increase in transcription, or even a repression of transcription. This is consistent with the report in the literature that histone substitutions have the greatest effect on transcriptional silencing^12^. Second, we observed increased occupancy at promoters in mutants H3K18A, H4K20A, H4K5A, and H4K16A and these mutants share the greatest number of common dynamic genes (Table S4, Fig. 4c and Fig. S9a). Moreover, the common genes are enriched in Gene Ontology terms of cellular components of cell wall and pathways of glycolysis and ribosomes. This common feature is probably due to the co-occurrence of acetylation on modifiable histone sites at promoters (Fig. S8)^16^,^26^. Third, genomic regions that are marked with trimethylation at H4K20 and acetylation at H4K16 in wild-type yeast are more dynamic upon histone mutation, whereas regions marked with acetylation of H3K18 tend to be more stable in the mutants (Fig. S2). Importantly, this effect is seen almost across all mutants and is independent of the mutation type, suggesting a similar cellular mechanism in response to histone mutation.

## Methods

### Yeast strains and culture conditions

The yeast histone mutant strains were derived from the SHIMA library, which is a complete library of alanine mutants at all residues of the four core histones, excluding the naturally occurring alanine residues, in the yeast *S. cerevisiae*^48^. Plasmids bearing alanine mutations were transformed into the yeast histone shuffle strains. Yeast mutant stains used in this study are listed in Table S1. Genotypes of the mutant strains are listed in Table S2. The H3 and H4 mutants were cultured in nutrient-defined medium lacking tryptophan (SC-Trp), and the H2A and H2B mutants were cultured in SC-His medium at 30 °C to select for plasmid maintenance. Mutants and wild-type strains were typically grown to an optical density at 600 nm (OD_600_) of 1.0. Each histone mutant strain was sequenced to confirm the mutation before use in experiments.

### Determination of nucleosome positioning

Wild-type cells were grown in yeast extract, peptone, and dextrose medium to an OD_600_ of 1.0 and transferred to SCIM medium for 8 h. All cells were then treated with formaldehyde and incubated with 125 mM glycine. Cell permeabilization, micrococcal nuclease digestion, protein degradation, and DNA purification steps were performed as previously described^49^. DNA samples were treated with RNase A and separated on a 2% agarose gel to assess the nucleosomal content. Bands corresponding to mononucleosomal DNA were extracted using a Qiagen Gel Extraction kit (Qiagen). Mononucleosomal DNA libraries were prepared and sequenced using an Illumina Genome Analyzer II (Illumina Inc. USA) according to the manufacturer’s instructions.

### Reverse transcription polymerase chain reaction (RT-PCR)

Extraction of total RNA and cDNA synthesis were carried out using standard methods^50^. Briefly, total RNA was isolated using Trizol reagent (Sigma). Total RNA (1 μg) was reverse transcribed to cDNA using PrimeScript RT-polymerase (Thermo Scientific) in a 20-μL reaction system. RT-PCR was performed using SYBR Green Mix (Roche), and relative expression levels were normalized to the level of β-actin. RT-PCR was carried out using a CFX96™ Real-Time System (Bio-Rad), and the quantitative RT-PCR data were analysed by the Δ*C*_t_ method as previously described^51^.

### Spot assays under stress conditions

Spot assays were performed in the presence of 0.05 M HU, 0.1 M HU, 0.2 M HU, 0.005% MMS, 0.0125% MMS, 0.03% MMS, 0.1 mM H_2_O_2_, 1 M NaCl, and glycerol stress conditions and in yeast extract, peptone, and dextrose medium at 30 °C, 33 °C, and 37 °C for temperature sensitivity tests. To determine growth defects by spot dilution assays the plates were photographed after incubation at 30 °C for 72 h for various stress conditions, or at the appropriate temperature for the temperature sensitivity tests.

### Nucleosome occupancy

The raw sequencing reads for nucleosome occupancy were mapped onto the *S. cerevisiae* strain S288c genome using Bowtie^52^. Only the uniquely mapped reads were used in further analysis. First, the length of each read was extended 73 bp in the 3′ direction, and the Watson-strand reads and Crick-strand reads were oppositely shifted 73 bp. The absolute nucleosome occupancy value of each genomic site was expressed as the number of reads covering the genomic sites. Second, nucleosome occupancy was scaled by dividing the occupancy value by the average nucleosome occupancy of the whole genome; i.e., the nucleosome occupancy was expressed as the fold change of the absolute occupancy relative to the average occupancy. It is necessary to indicate that there are no internal controls to compare occupancy between two different strains^47^, therefore an alteration in nucleosome occupancy is relative to all other nucleosomes in the same strain.

### Nucleosome occupancy profiles

The nucleosome occupancy profiles near special sites (e.g., TSSs) were represented by the average nucleosome occupancy profile, which was calculated by summing the occupancy signal at each genomic site and then dividing the summed signal by the gene number^53^. Nucleosome occupancy was represented as the difference in occupancy between the mutant and wild-type strains (mutant − wild type).

### Identification of nucleosome dyad position

The dyad coordinates of nucleosomes were identified with a wavelet transformation-based peak-finding algorithm (the “peaksfind” function in MATLAB [R2009]). The nucleosome occupancy profile was smoothed using a Daubechies wavelet at level 4. Peaks that satisfied the following two criteria were then identified: (i) height >1.2 (i.e., occupancy is 1.2-fold higher than the average occupancy), and (ii) full width at half height ≥73 bp.

A matching percentage was calculated to indicate the degree of matching of nucleosome dyad positions between two biologic replications or between the mutant and wild-type strains. Given a deviation, the matching percentage represents the number of matched nucleosomes between two strains relative to the total number of identified nucleosomes in the first experimental replication (Rep1) or wild type, as appropriate. A matched nucleosome means that the difference in the dyad positions between two strains is less than the deviation. The deviation ranged from 1 bp to 60 bp.

### Genome-wide dynamics of nucleosome occupancy

The genome-wide dynamics of nucleosome occupancy were identified as follows. First, the genome was divided into continuous 0.5-kb segments, resulting in 24,135 segments covering the whole genome. Second, for each segment, a correlation coefficient was calculated for the nucleosome occupancy between the mutant and wild-type strains. If the correlation coefficient was less than 0.5, the nucleosome occupancy was considered to be dynamic in the segment; if the correlation coefficient was greater than 0.5, the nucleosome occupancy was considered to be stable. For a dynamic segment, if the average nucleosome occupancy in the mutant strain was greater than that in the wild-type strain, we concluded that the nucleosome occupancy was increased in the mutant strain; otherwise, the nucleosome occupancy was decreased in the mutant strain.

### Nucleosome occupancy dynamics at promoters

We retrieved the genomic coordinates of both ends of 5,419 transcripts from the *Saccharomyces* Genome Database (http://www.yeastgenome.org/). Nucleosome occupancy dynamics at promoter regions (−0.5 kb to 0.3 kb) were also examined using the correlation coefficient between the mutant and wild-type strains. A low coefficient, less than 0.5, indicates greater alterations in nucleosome occupancy. The coefficient was calculated for 5,419 genes in 22 mutants. For each mutant, the top 108 dynamic genes and the top 108 stable genes were chosen for further analysis. The transcription rates were compared between the dynamic genes and the stable genes. Data on the transcription rates of yeast genes were obtained from a previous study^54^.

### Dataset of histone modification

We downloaded a dataset of 14 types of histone modifications in wild-type strains from the literature (Gene Expression Omnibus [GEO] accession number: GSE61888)^16^. Mapping the reads was performed using parameters from the literature and the calculated profile of histone modifications was similar to the nucleosome occupancy profile.

### Enrichment analysis

Enrichment analysis was carried out for KEGG pathway and GO term data using the functional annotation table module of DAVID (http://david.abcc.ncifcrf.gov/).

## Additional Information

**How to cite this article**: Liu, H. *et al.* Nucleosome alterations caused by mutations at modifiable histone residues in *Saccharomyces cerevisiae*. *Sci. Rep.* **5**, 15583; doi: 10.1038/srep15583 (2015).

## Supplementary Material

Supplementary Information
