# Supplementary material for: Nucleosome alterations caused by mutations at modifiable histone residues in Saccharomyces cerevisiae
Source: Sci Rep. 2015 Oct 26;5:15583. doi: 10.1038/srep15583 (PMC4620441; doi:10.1038/srep15583)
Supplement: Supplementary Information [file srep15583-s1.pdf]

Nucleosome alterations caused by mutations at modifiable histones residues in *Saccharomyces cerevisiae*

Hongde Liu[1,+,*], Pingyan Wang[2,+], Lingjie Liu[1], Zhu Min[2,4], Kun Luo[3] and Yakun Wan[2,4,*]

[1]State Key Laboratory of Bioelectronics, Southeast University, Nanjing 210096, China;
[2]Institute of Life Sciences, Southeast University, Nanjing 210096, China;
[3]Department of Neurosurgery, Xinjiang Evidence-Based Medicine Research Institute, First Affiliated Hospital of Xinjiang Medical University, Urumqi 830054, China;
[4]Shanghai Institute of Materia Medica, Chinese Academy of Sciences, Shanghai 201203, China

[+]These authors contributed equally to this work.
*Corresponding authors: liuhongde@seu.edu.cn (HDL); ykwan@simm.ac.cn (YKW).

E-mail:
liuhongde@seu.edu.cn (HDL)
yami_seu@163.com (PYW)
lingjie26@126.com (LJL)
zhumin555111@163.com (ZM)
luokun_2822@sohu.com (KL)
ykwan@simm.ac.cn (YKW)

Running title: Nucleosome dynamics and histone mutations

Supporting information

Table captions

Table S1 Yeast histone mutants used in this study.

Table S2 Genotypes of the relevant strains.

Table S3 Percentage of dynamic genomic regions in 22 mutants
The whole yeast genome was divided into 24,135 continuous segments. Each segment was 0.5 kb in length. A dynamic segment was identified if the correlation coefficient of the nucleosome occupancy between the mutant and wild-type strains was less than 0.5 (see Methods). The table lists the percentage of dynamic segments relative to all segments. For any segment, if the average nucleosome occupancy in the mutant strain is greater than that in the wild-type strain, the nucleosome occupancy is increased in the segment upon histone mutation; otherwise, the nucleosome occupancy is decreased. The 22 mutants were ranked in the fifth column by the percentage of dynamic segments. The distribution of the dynamic segments is shown in Figure S1a.

Table S4 Hypothesis test for the promoter distribution in the nucleosome occupancy-increased and occupancy-decreased genomic segments.
The test is based on hypergeometric distribution. The yeast genome was divided into 24,135 continuous segments where there are 6253 promoter-segments. The promoter-segments mean that the segments centers are at the promoters (the genomic regions of −0.5 kbp ~ +0.3 kbp relative to TSSs). The nucleosome occupancy -increased and -decreased segments were counted respectively. Also, the occupancy -increased and -decreased promoter-segments were counted respectively. The hypothesis test was carried out for both occupancy -increased and -decreased promoter-segments.
Significance was indicated with P-value ($-\log_{10}$) of the test. The occupancy-increased segments were significantly enriched at promoters in mutants H3S10A, H3K18A, H4K20A, H4K5A and H4K16A. Contrastingly, the occupancy-decreased segments were not enriched at promoters in any mutants.

Figure legends

Figure S1 Different histone mutations cause different alterations in the nucleosome occupancy of the whole genome.

(a): Distribution (pie plot) of the dynamic segments for each mutant. The dynamic segments ($r < 0.5$) are counted by their genomic loci: promoter (−0.5 kb to 0.3 kb relative to the transcription start site [TSS]), gene body (0.3 kb to TTS), and other regions.

(b): Number of overlapped dynamic segments between the mutants (lower triangular matrix) and the ratio (R) of the percentage of the overlapped dynamic segments in mutant A to the percentage of the dynamic segments in mutant B to total segments (24,135) (upper triangular matrix).

Given the number (O_num) of overlapped dynamic segments between mutant A and mutant B, and the number of the dynamic segments in the mutant A (A_num) and the mutant B (B_num), respectively, the ratio (R) was calculated with R=[O_num/A_num]/[B_num/24135], which indicating the overlapped degree between two mutants. The result was shown in the upper triangular matrix. We got infinitesimal P-values for all pairs of the mutants in using hypergeometric test to test the significance of the overlapped dynamic segments. Thus, instead of P-values, we used the ratio (R) to indicate the overlapped degree. The results indicated that the dynamic segments are highly overlapped between the pairs of the mutants H3K9A and H4R3A, the mutants H2B123A and H2B16A, and the mutants H2B16A and H2AS128A.

Figure S2 The correlation coefficients (r) between histones modifications and nucleosome dynamics.

The yeast genome was divided into 24,135 continuous segments (see Method section and Fig. 1). Each segment was 0.5 kb in length. The nucleosome dynamics of each segment between the mutant and wild-type strain was indicated with correlation coefficient (Nu-r). We firstly calculated the average value (av-HM) of histone modification profile at each segment. Then, we calculated correlation coefficient (r) between the average value of histone modification (av-HM) and the nucleosome dynamics (Nu-r) for the 24,135 segments. The coefficient (r), shown in the table, indicates the association between the histone modification and nucleosome dynamics. A greater coefficient (r) suggests that the histone modification associates nucleosome unstability (dynamics); reversely, a smaller coefficient (r) suggests that the histone modification is favorable to nucleosome stability.

Acetylations at H3K14, H3K18, H4K5 and H4K8 (H3K4Ac, H3K18Ac, H4K5Ac and H4K8Ac) prefer nucleosome-stable genomic segments. Tri-methylation at H4K20 (H4K20Me3) and acetylation at H4K16 prefer nucleosome-dynamic genomic segments. The effects are found almost in all of the mutants, suggesting that the effects are independent with the types of histones mutations.

Figure S3 Nucleosome occupancy difference and histone modification profile at telomeres and centromeres.

(a): Difference significance of nucleosome occupancy at telomeres and centromeres between each mutant and the wild-type strain. The bar plot shows the P-values ($-\log_{10}$) that were calculated

with a two-sample *t*-test for the average nucleosome occupancy in telomeres between each mutant and wild-type strain. P-values for centromeres are also shown.
(b): Histones modifications at telomeres and centromeres in wild type strain. The profile of histone modification is represented with relative reads counts (fold change of reads count at each genomic region to average reads count of whole genome). Shown profiles are averaged for 16 chromosomes.

Figure S4 Variation of occupancy and dyad position of nucleosomes near transcription start and terminations sites (TSSs and TTSs).
(a): Dyad position shift of the mutant to the wild type (left panels) and occupancy variation (right panels). The dyad position was identified with function "peaksfind()" of Matlab (R2009) (see Method section).
(b): Difference significance of occupancy at the -1, +1 and +2 nucleosomes and in 400-bp region around TTSs between the mutant and the wild type strain. Shown is $-\log_{10}$(P-values) of two-sample *t*-test.

Figure S5 Nucleosome occupancy dynamics of 5419 genes.
(a): Nucleosome occupancy profiles in the vicinity of TSSs for 5419 genes in 22 mutant strains.
(b): Dynamics of the −1 and +1 nucleosomes at 5419 promoters. Nucleosome occupancy dynamics are defined by five groups: stable, position shift < 30 bp and −2 < fold change of occupancy < 2; increased, position shift < 30 bp and fold change of occupancy > 2; decreased, position shift < 30 bp and fold change of occupancy < −2; gain, position shift > 147 bp and fold change of occupancy > 2; and loss, position shift > 147 bp and fold change of occupancy < −2. The table indicates the number of each dynamic type for the −1 and +1 nucleosomes of 5419 promoters in 22 mutants.

Figure S6 Fourier spectra of nucleosome occupancy in the mutant and wild-type strains.
First, the autocorrelation signal of the nucleosome occupancy profile of 16 chromosomes was calculated. Then, a fast Fourier transformation (FFT) was performed using the autocorrelation signal.

Figure S7 Clustering of correlation coefficients.
For each of the 5419 yeast genes, the correlation coefficient of the nucleosome occupancy profile in the range of −0.5 kb to 0.3 kb relative to the TSS was calculated between each mutant strain and the wild-type strain. This was done for 22 mutants, resulting in matrix of 5419 × 22. The bi-clustering for the matrix is shown.

Figure S8 Profiles of histones modifications around transcription start sites of both top 108 dynamic genes and top 108 stable genes.
The dynamic genes and the stable genes were identified by calculating correlation coefficients of nucleosome occupancies between the mutant and the wild type strain (see Method section and Fig. 3).

Figure S9 Common dynamic genes in mutant strains

(a): The number of overlapping dynamic genes in mutants H3S10A, H3K56A, H4K16A, H4K20A, H3K18A, and H4K5A. In each mutant strain, dynamic genes are identified by ranking the correlation coefficients of the nucleosome occupancy profiles between the mutant and wild-type strains (see Fig. 3 and Methods). The 108 genes with the lowest coefficients were chosen. The table indicates the number of overlapping dynamic genes.

(b): Same as A except for overlapping stable genes. For each mutant, the top 108 stable genes that are associated with the highest coefficients were chosen.

(c): Nucleosome occupancy profiles for the *TDH2* gene in the H3S10A, H3K56A, H4K16A, and H4K20A mutant strains. The black line indicates the wild-type profile.

(d): Same as C except for the *CDC19* gene.

(e): Same as C except for the *RPS16A* gene.

Figure S10 Enrichment analysis for Gene Ontology terms for the top 108 dynamic genes in each mutant strain. The analysis was performed using DAVID.

Figure S11 Western blot analysis of histone H3 in wild-type strain and histone mutant strain. 108 cells of each strain were harvested and homogenized in protein extraction buffer (20 mM Tris PH 7.5, 0.1M NaCl, 1 mM EDTA, 1× protease inhibitor cocktail (PIC)) to prepare of whole protein extracts. The lysates were centrifuged at 12,000× g at 4 ° C for 2 min. Protein concentrations were determined by the BCA protein assay kit and 120 μ g of protein were loaded per well on SDS-PAGE. Subsequently, proteins were transferred to PVDF membrane (0.22 μ M, Millipore, USA). Membrane was blocked at room temperature for 1 h with 5% skimmed milk in Tris-buffered solution plus Tween-20 (TBST). Membrane was then incubated overnight at 4 ° C with primary antibodies for Anti-Histone H3 (Millipore, USA). After three 10 min washing in TBST, membrane was incubated for 2 h at room temperature with a horseradish peroxidase-conjugated secondary antibody. The proteins were visualized using an enhanced chemiluminescence Western blot detection kit. The relative expression of H3 proteins was quantified by the Image-Analysis system.

Figure S12 Test of biological replication for the mutants H3S10A, H3K56A, H4K20A and H4K16A.

(a): Nucleosome occupancy profiles for a segment of chromosome 1 in two biological replications (Rep1 and Rep2) for the four types of mutant strains.

(b): the nucleosome occupancy at both the telomeres and the centromeres in two biological replications. Top panels are for replication 1, and bottom panels are for replication 2.

Table S1

Yeast histone mutants used in this study

H2A(HTA1)	H2B(HTB1)	H3(HHT2)	H4(HHF2)
K21A	K16A	K4A	R3A
S121A	K123A	K23A	K20A
S128A		K14A	K5A
K7A		K36A	K91A
		S28A	K16A
		K79A	K8A
		K9A	
		S10A	
		K56A	
		K18A	

Table S2

Genotype of the relevant strains

Strain	Genotype
YBL574	*MAT* **a** *leu2-1 his3-200 ura3-52 trp1-63 lys2-128 (hht1-hhf1)::LEU2 (hht2-hhf2)::HIS3 Ty912Δ35-lacZ::HIS4 pWA414 [F12-HHT2-HHF2]*
FY406	*MAT* **a** *leu2-1 his3-200 ura3-52 trp1-63 lys2-128 (hta1-htb1)::LEU2 (hta2-htb2)::TRP1 pZS145 [HTA1-Flag-HTB1]*

Table S3

Percentage of dynamic genomic regions in 22 mutants

		**Percentage of dynamic regions** (total: 24135 0.5kbp-long genomic segments) (100%)	**Percentage of nucleosome-increased regions (P_up)** (100%)	**Percentage of nucleosome-decreased regions (P_down)** (100%)	**Rank**
**H2A**	K21A	3.8742	1.4171	2.4571	16
	S121A	4.6698	1.2182	3.4516	15
	S128A	2.5524	0.7458	1.8066	17
	K7A	1.9143	0.7003	1.2141	18
**H2B**	K16A	1.1975	0.3895	0.8080	21
	K123A	0.7210	0.3108	0.4102	22
**H3**	K4A	7.1062	3.0123	4.0938	10
	K23A	4.8769	2.1173	2.7596	14
	K14A	5.8631	2.5897	3.2734	12
	K36A	14.0797	7.8147	6.2650	6
	S28A	5.4985	2.0220	3.4764	13
	K79A	14.5314	6.2609	8.2705	4
	K9A	1.7651	0.7707	0.9944	19
	S10A	10.8312	5.6601	5.1711	8
	K56A	12.7952	5.4156	7.3796	7
	K18A	9.7912	4.4129	5.3783	9
**H4**	R3A	1.3964	0.7044	0.6920	20
	K20A	15.4802	6.1490	9.3312	1
	K5A	14.7220	5.1794	9.5426	3
	K91A	14.4361	6.5675	7.8686	5
	K16A	15.2813	6.7581	8.5232	2
	K8A	6.0910	2.2831	3.8079	11

Table S4

Hypothesis test for the promoter distribution in the nucleosome occupancy-increased and occupancy-decreased genomic segments

Mutants strains	Hyper-geometric test for the promoter distribution in the nucleosome occupancy-increased genomic segments				Hyper-geometric test for the promoter distribution in the nucleosome occupancy-decreased genomic segments			
	Number of the occupancy-increased segments (SI_N)	Number of promoters in the occupancy-increased segments (PI_N)	[PI_N] /[SI_N]	P-value ($-\log_{10}$)	Number of the occupancy-decreased segments (SD_N)	Number of promoters in the occupancy-decreased segments (PD_N)	[PD_N] /[SD_N]	P-value ($-\log_{10}$)
H2AK21A	342	14	0.0410	0	593	116	0.1960	0
H2AS121A	294	74	0.2520	0.2000	833	21	0.0250	0
H2AS128A	180	35	0.1940	0	436	39	0.0890	0
H2AK7A	169	33	0.1950	0	293	34	0.1160	0
H2BK16A	94	12	0.1280	0	195	22	0.1130	0
H2BK123A	75	6	0.0800	0	99	2	0.0200	0
H3K4A	727	94	0.1290	0	988	50	0.0510	0
H3K23A	511	53	0.1040	0	666	55	0.0830	0
H3K14A	625	80	0.1280	0	790	31	0.0390	0
H3K36A	1886	124	0.0660	0	1512	40	0.0260	0
H3S28A	488	38	0.0780	0	839	118	0.1410	0
H3K79A	1511	226	0.1500	0	1996	94	0.0470	0
H3K9A	186	68	0.3660	3.3000	240	17	0.0710	0
H3S10A	1366	624	0.4570	Inf	1248	93	0.0750	0
H3K56A	1307	384	0.2940	2.8000	1781	122	0.0690	0
H3K18A	1065	401	0.3770	Inf	1298	62	0.0480	0
H4R3A	170	43	0.2530	0.3000	167	17	0.1020	0
H4K20A	1484	656	0.4420	Inf	2252	203	0.0900	0
H4K5A	1250	517	0.4140	Inf	2303	280	0.1220	0
H4K91A	1585	175	0.1100	0	1899	123	0.0650	0
H4K16A	1631	712	0.4370	Inf	2057	153	0.0740	0
H4K8A	551	82	0.1490	0	919	42	0.0460	0

Figure S1

(a)

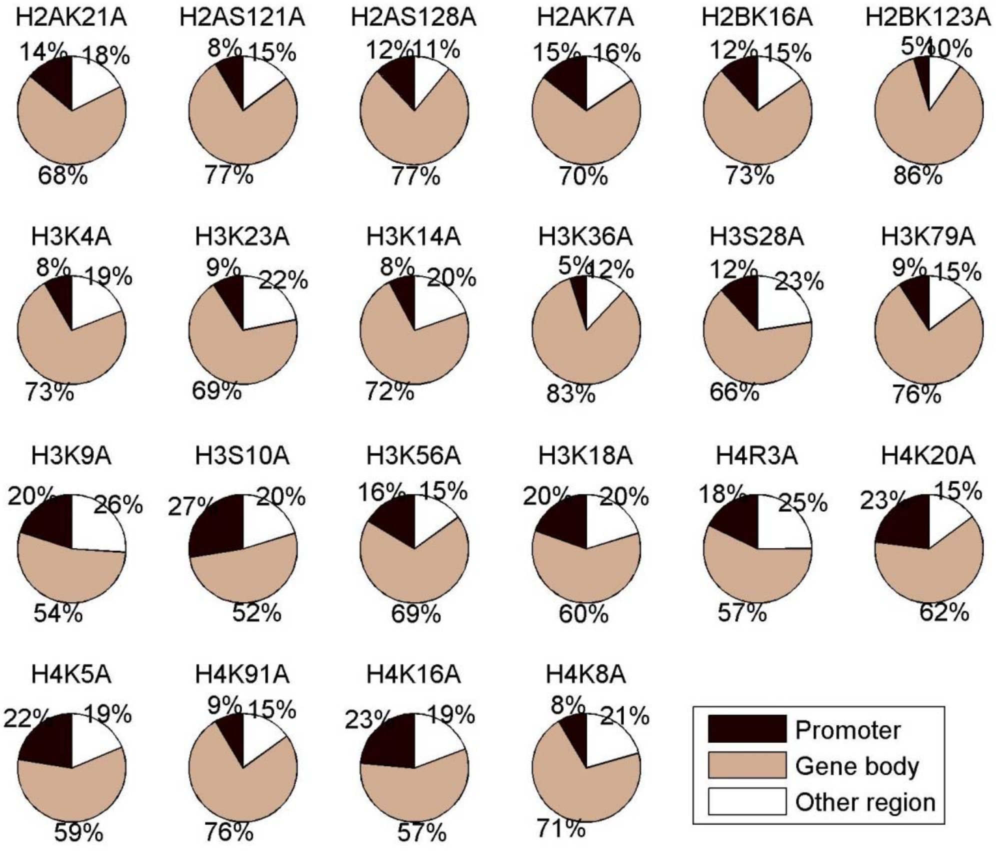

(b)

H2AK21A	H2AS121A	H2AS128A	H2AK7A	H2BK16A	H2BK123A	H3K4A	H3K23A	H3K14A	H3K36A	H3S28A	H3K79A	H3K9A	H3S10A	H3K56A	H3K18A	H4R3A	H4K20A	H4K5A	H4K91A	H4K16A	H4K8A	
935	4.5	8.9	8	10.4	7.9	6.1	7.3	6.7	3.9	7.1	3.8	7.1	3.3	2.8	4.6	7.6	2.5	3.4	3.8	3.2	6.1	H2AK21A
195	1127	7.5	8.6	11.6	10.3	4.8	5.1	4.9	3.4	4.3	3.4	7.2	3.8	4.9	3.9	7.6	3.7	2.7	3.2	3.3	4.9	H2AS121A
213	217	616	13.4	16.9	10.4	5.9	6.5	6.6	4	6.2	4.2	10.4	3.9	3.4	4.6	10.9	3	3.4	4	3.3	6.2	H2AS128A
143	185	158	462	21.5	13.5	6.5	8	7.6	4.1	7.8	4.1	12.9	4.7	4.2	5.3	14.1	3.2	3.7	3.9	3.8	7.3	H2AK7A
116	156	125	119	289	17.8	7.2	8.7	7.8	4.3	7.8	4.2	13.7	4.2	4.1	5.4	16.1	3.5	4	4	3.8	7.2	H2BK16A
53	84	46	45	37	174	6.7	6.1	6.8	4.9	6.7	4.2	9.4	3.3	4.6	4.2	13.6	3.2	3	4.1	3.3	6.4	H2BK123A
406	381	260	212	147	83	1715	9.8	9.3	5.3	8.5	5.1	7	3.7	3	5.8	7.8	2.6	3.8	4.7	3.9	9	H3K4A
334	280	194	180	123	52	818	1177	11.5	5.1	11.5	4.9	9.6	3.8	3	6.3	9.2	2.8	4.2	4.9	4.4	10.9	H3K23A
370	323	238	207	132	69	937	791	1415	5	9.8	4.9	8.3	3.6	3	6	8.9	2.5	3.8	4.7	3.9	9.6	H3K14A
509	541	347	265	176	120	1280	845	1004	3398	4.5	4.1	4	2.5	2.7	3.6	4.5	2.1	2.5	3.9	2.7	5.1	H3K36A
366	265	209	199	124	64	802	742	761	844	1327	4.5	8.8	3.9	2.7	6.1	8.7	2.6	4.4	4.6	4.1	9.6	H3S28A
512	562	380	273	177	106	1259	835	1011	2023	865	3507	4.3	3	2.8	3.8	4.7	2.4	2.7	3.7	3.1	5.2	H3K79A
117	143	113	105	70	29	213	199	207	241	205	266	426	6.6	5.6	7.1	19	5	5.2	4	5.1	9.6	H3K9A
337	466	259	233	131	63	687	487	546	906	554	1131	306	2614	4	5.1	5.6	4.1	4.1	2.5	4.6	4.3	H3S10A
339	702	270	247	152	102	666	451	544	1171	455	1274	304	1328	3088	3	5.1	3.7	2.3	2.4	2.9	3.2	H3K56A
425	425	276	240	152	72	972	730	826	1185	798	1314	296	1312	921	2363	5.7	3	5.1	3.6	4.7	6.7	H3K18A
99	119	94	91	65	33	187	151	175	213	162	231	113	206	221	189	337	3.9	4.1	4.3	4.1	8	H4R3A
366	640	289	231	158	86	680	506	550	1126	530	1303	327	1655	1753	1081	202	3736	2.4	2	3.4	2.9	H4K20A
473	454	308	250	172	77	962	722	797	1266	863	1388	326	1567	1047	1762	202	1305	3553	2.6	3.7	4.5	H4K5A
510	517	355	261	168	102	1164	827	962	1935	876	1850	248	950	1080	1212	210	1083	1359	3484	2.7	4.9	H4K91A
461	564	311	270	166	89	1018	789	844	1412	835	1644	335	1849	1356	1699	209	1920	2007	1437	3688	4.6	H4K16A
346	334	232	205	127	68	939	782	829	1050	776	1111	250	686	594	961	164	671	972	1046	1039	1470	H4K8A

Figure S2

	H3K4Ac	H3K23Ac	H3K14Ac	H3K36Me3	H3K79Me3	H3K9Ac	H3S10Ph	H3K56Ac	H3K18Ac	H4R3Me3	H4K20Me3	H4K5Ac	H4K16Ac	H4K8Ac
H2AK21A	0.079	0.067	0.121	-0.047	-0.012	0.049	-0.002	0.031	0.127	-0.058	-0.081	0.092	-0.061	0.079
H2AS121A	0.062	0.054	0.12	-0.086	-0.03	0.014	-0.034	-0.001	0.126	-0.123	-0.149	0.164	-0.138	0.145
H2AS128A	0.084	0.068	0.125	-0.084	-0.03	0.049	-0.012	0.028	0.139	-0.085	-0.127	0.127	-0.101	0.109
H2AK7A	0.095	0.089	0.151	-0.061	-0.019	0.057	-0.003	0.035	0.158	-0.075	-0.1	0.136	-0.077	0.118
H2BK16A	0.102	0.091	0.154	-0.051	-0.001	0.061	0.004	0.043	0.146	-0.068	-0.087	0.132	-0.066	0.12
H2BK123A	0.143	0.12	0.212	-0.066	0.013	0.091	-0.012	0.058	0.221	-0.104	-0.136	0.164	-0.101	0.154
H3K4A	0.081	0.067	0.138	-0.093	-0.037	0.037	-0.026	0.017	0.149	-0.103	-0.134	0.105	-0.112	0.093
H3K23A	0.068	0.055	0.12	-0.085	-0.037	0.027	-0.025	0.012	0.13	-0.096	-0.125	0.101	-0.108	0.088
H3K14A	0.066	0.053	0.12	-0.098	-0.045	0.024	-0.029	0.009	0.13	-0.101	-0.129	0.087	-0.112	0.075
H3K36A	0.109	0.079	0.169	-0.127	-0.019	0.055	-0.032	0.031	0.193	-0.13	-0.187	0.126	-0.155	0.115
H3S28A	0.088	0.075	0.14	-0.061	-0.02	0.051	-0.012	0.031	0.149	-0.075	-0.096	0.098	-0.075	0.087
H3K79A	0.07	0.05	0.116	-0.121	-0.048	0.029	-0.041	0.007	0.137	-0.119	-0.161	0.101	-0.137	0.084
H3K9A	0.085	0.082	0.137	-0.031	-0.005	0.051	0.005	0.034	0.14	-0.058	-0.072	0.127	-0.06	0.112
H3S10A	0.078	0.1	0.139	0.074	0.039	0.054	0.033	0.036	0.092	-0.013	0.013	0.177	0.025	0.157
H3K56A	0.069	0.072	0.119	-0.021	-0.009	0.035	-0.007	0.019	0.106	-0.069	-0.078	0.158	-0.061	0.137
H3K18A	0.094	0.096	0.16	-0.009	0.007	0.059	0.005	0.035	0.145	-0.056	-0.057	0.139	-0.038	0.124
H4R3A	0.149	0.139	0.209	-0.001	0.035	0.111	0.033	0.086	0.209	-0.032	-0.037	0.147	-0.014	0.135
H4K20A	0.008	0.029	0.055	-0.013	-0.029	-0.018	-0.012	-0.024	0.029	-0.062	-0.074	0.157	-0.071	0.13
H4K5A	0.114	0.117	0.173	0.044	0.048	0.089	0.032	0.062	0.147	-0.014	0	0.135	0.022	0.125
H4K91A	0.052	0.027	0.089	-0.147	-0.073	0.021	-0.041	0	0.128	-0.108	-0.174	0.068	-0.155	0.054
H4K16A	0.026	0.046	0.086	-0.002	-0.02	-0.002	-0.012	-0.016	0.06	-0.067	-0.066	0.156	-0.059	0.133
H4K8A	0.074	0.062	0.13	-0.093	-0.042	0.031	-0.027	0.013	0.141	-0.101	-0.133	0.104	-0.114	0.092

Corr. Coef. (r)

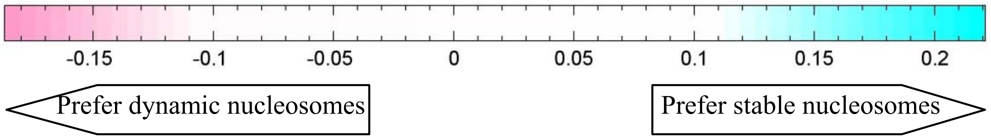

Figure S3

(a)

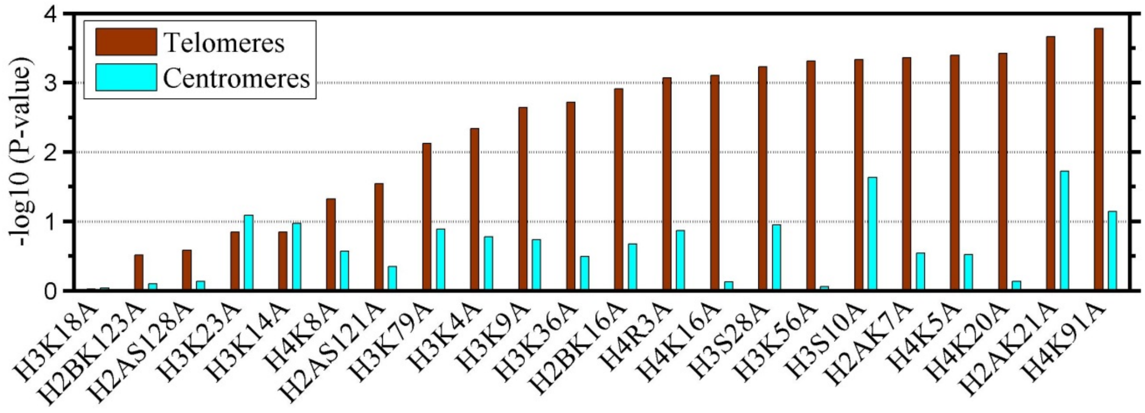
Telomeres
Centromeres
-log10 (P-value)
H3K18A
H2BK123A
H2AS128A
H3K23A
H3K14A
H4K8A
H2AS121A
H3K79A
H3K4A
H3K9A
H3K36A
H2BK16A
H4R3A
H4K16A
H3S28A
H3K56A
H3S10A
H2AK7A
H4K5A
H4K20A
H2AK21A
H4K91A

(b)

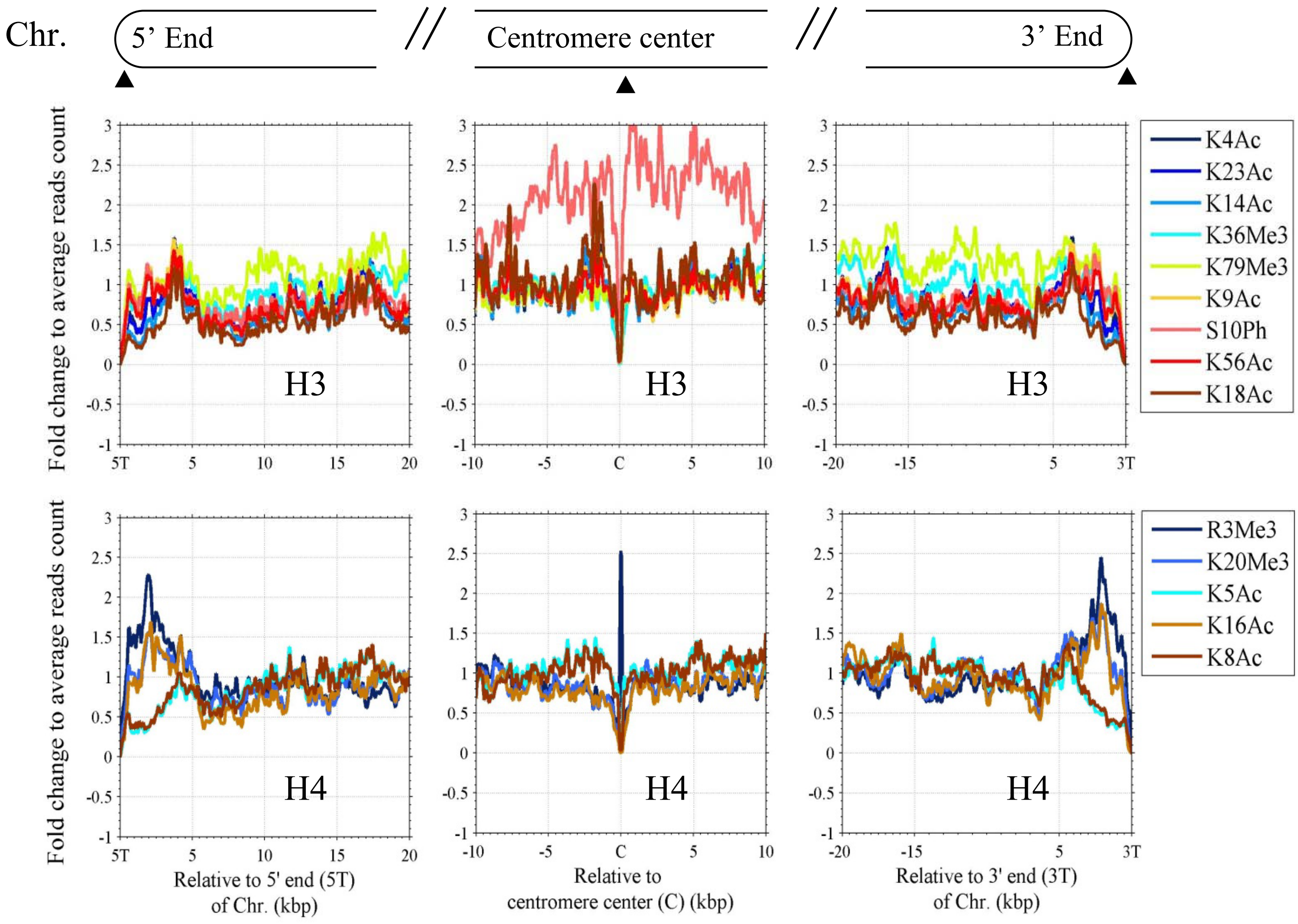
Chr.
5' End
Centromere center
3' End
Fold change to average reads count
H3
H4
K4Ac
K23Ac
K14Ac
K36Me3
K79Me3
K9Ac
S10Ph
K56Ac
K18Ac
R3Me3
K20Me3
K5Ac
K16Ac
K8Ac
Relative to 5' end (5T) of Chr. (kbp)
Relative to centromere center (C) (kbp)
Relative to 3' end (3T) of Chr. (kbp)

Figure S4

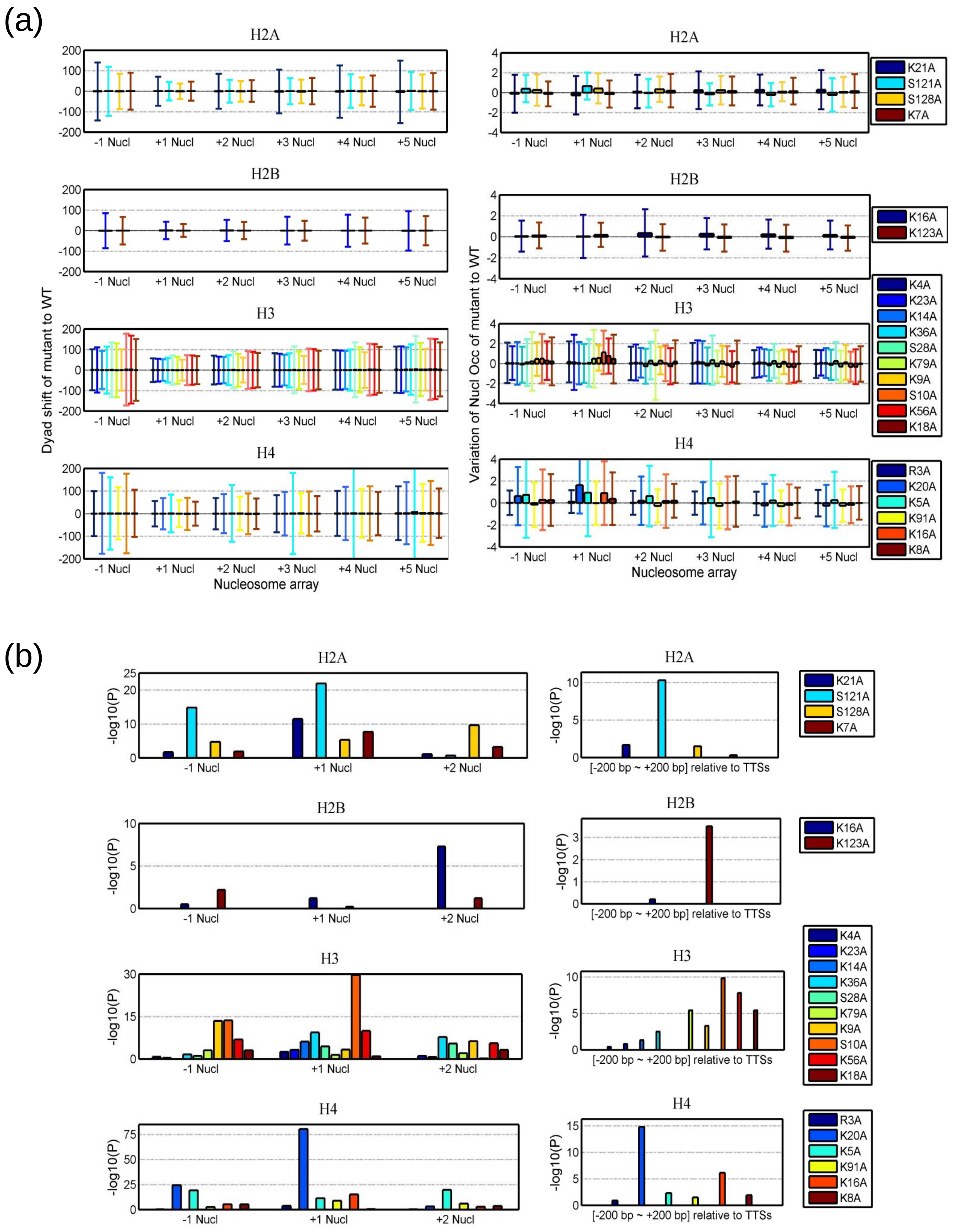
(a)
H2A
H2B
H3
H4
Dyad shift of mutant to WT
Variation of Nucl Occ of mutant to WT
-1 Nucl
+1 Nucl
+2 Nucl
+3 Nucl
+4 Nucl
+5 Nucl
Nucleosome array
K21A
S121A
S128A
K7A
K16A
K123A
K4A
K23A
K14A
K36A
S28A
K79A
K9A
S10A
K56A
K18A
R3A
K20A
K5A
K91A
K16A
K8A
(b)
-log10(P)
[-200 bp ~ +200 bp] relative to TTSs

Figure S5

(a)

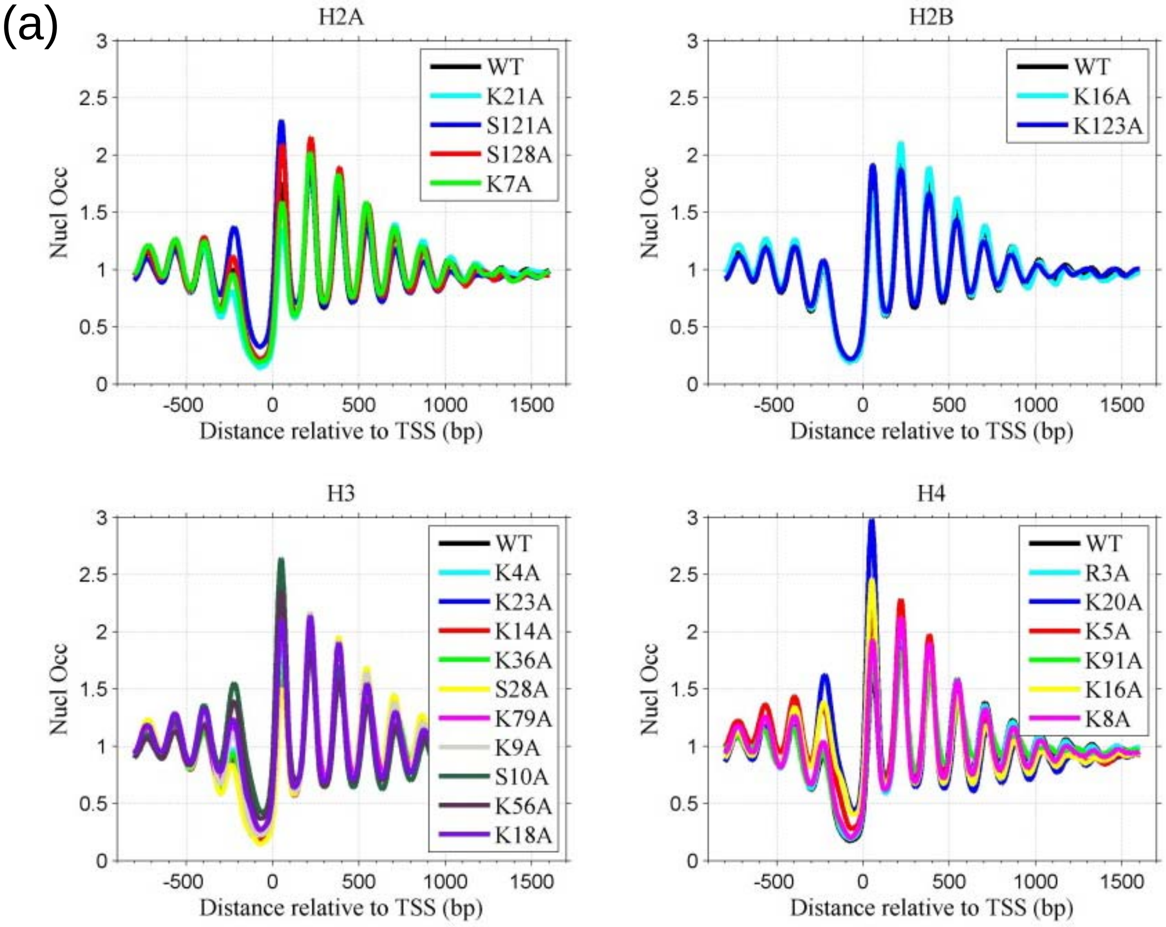

(b)

	The -1 nucleosome						The +1 nucleosome					
	Increase	Decrease	Left shift	Right shift	Loss	Gain	Increase	Decrease	Left shift	Right shift	Loss	Gain
H2AK21A	73	1093	123	143	74	62	63	1203	119	135	60	56
H2AS121A	814	31	87	94	86	85	685	45	71	91	36	35
H2AS128A	154	110	121	118	30	22	164	58	121	96	17	18
H2AK7A	96	446	98	101	20	22	77	471	103	95	18	27
H2BK16A	100	486	96	108	16	19	91	503	78	86	12	19
H2BK123A	107	9	87	92	18	15	63	5	60	49	14	15
H3K4A	413	161	169	161	24	27	449	159	125	135	28	25
H3K23A	194	305	136	113	21	26	211	301	121	104	27	27
H3K14A	315	230	153	138	21	20	317	219	125	118	28	27
H3K36A	433	189	180	138	32	33	507	195	146	114	35	39
H3S28A	172	644	134	120	37	39	156	692	125	105	44	29
H3K79A	871	133	190	146	69	55	1056	121	135	124	71	58
H3K9A	411	65	106	111	26	15	387	59	91	83	13	16
H3S10A	1042	52	129	116	352	352	1483	80	95	92	137	144
H3K56A	1117	95	115	134	230	222	1334	139	92	100	125	110
H3K18A	617	128	153	135	138	143	697	153	118	111	53	64
H4R3A	296	37	131	100	27	24	329	29	99	88	37	25
H4K20A	1297	67	162	130	340	359	2054	95	91	92	86	85
H4K5A	692	644	153	166	178	161	813	895	117	116	48	58
H4K91A	306	299	208	181	36	38	353	253	181	165	36	33
H4K16A	783	117	165	161	303	314	1192	177	123	118	78	88
H4K8A	440	201	145	140	24	22	456	175	132	106	22	31

Figure S6

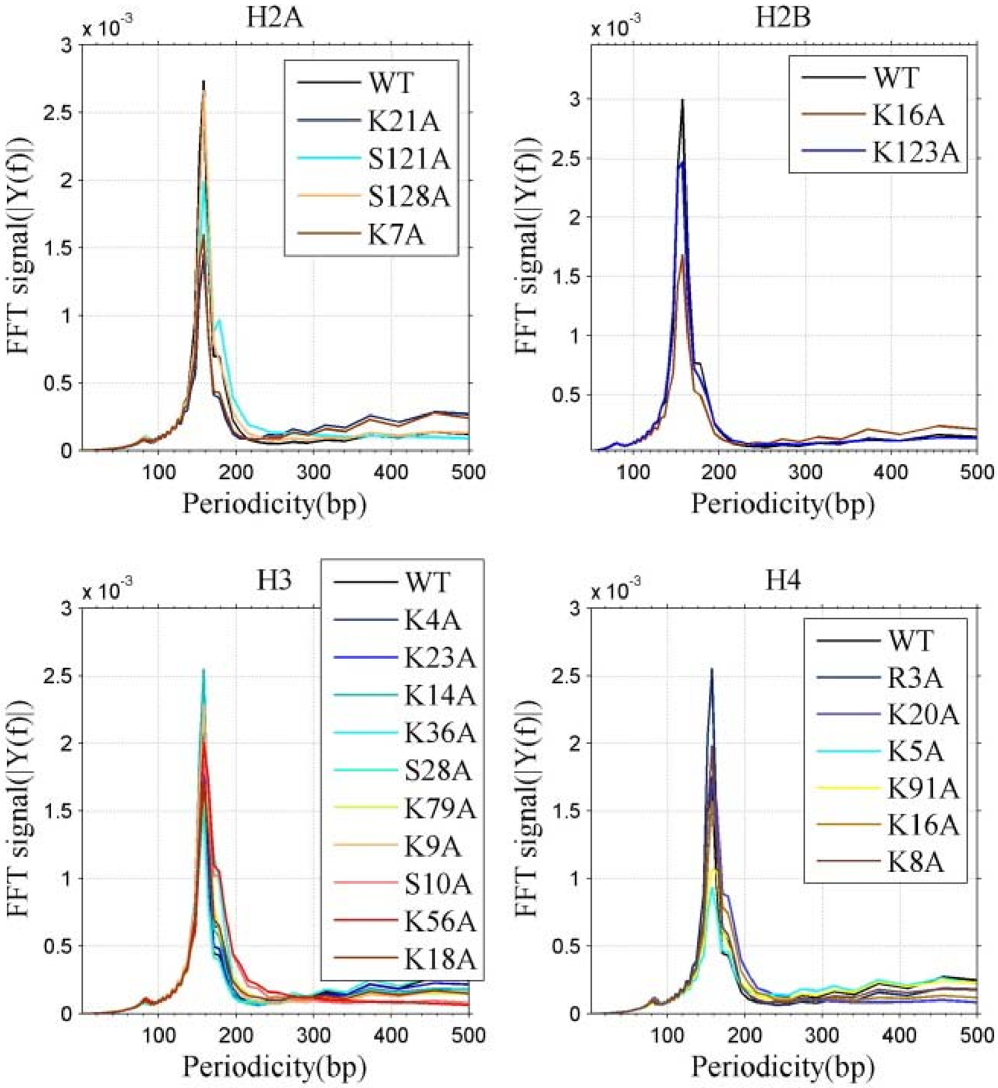

H2A
x 10^-3
WT
K21A
S121A
S128A
K7A
FFT signal(|Y(f)|)
Periodicity(bp)
H2B
WT
K16A
K123A
H3
WT
K4A
K23A
K14A
K36A
S28A
K79A
K9A
S10A
K56A
K18A
H4
WT
R3A
K20A
K5A
K91A
K16A
K8A

Figure S7

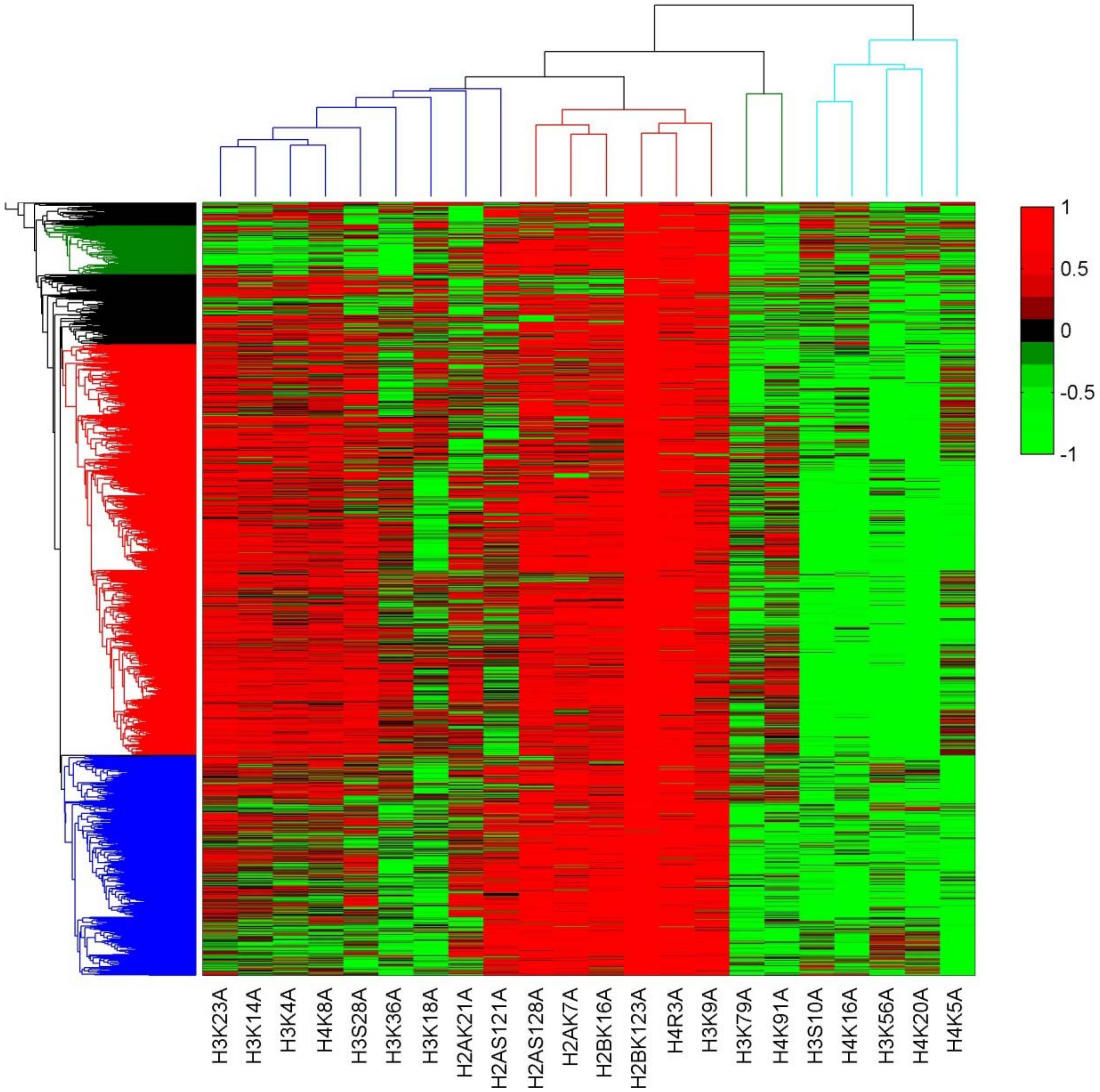
1
0.5
0
-0.5
-1
H3K23A
H3K14A
H3K4A
H4K8A
H3S28A
H3K36A
H3K18A
H2AK21A
H2AS121A
H2AS128A
H2AK7A
H2BK16A
H2BK123A
H4R3A
H3K9A
H3K79A
H4K91A
H3S10A
H4K16A
H3K56A
H4K20A
H4K5A

Figure S8

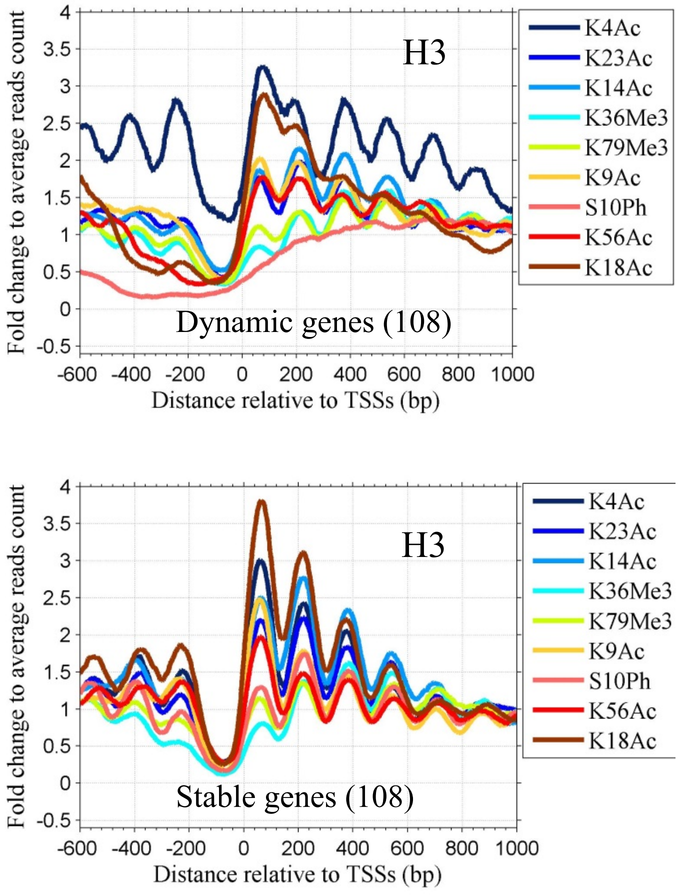
H3
H4
Dynamic genes (108)
Stable genes (108)
Fold change to average reads count
Distance relative to TSSs (bp)
K4Ac
K23Ac
K14Ac
K36Me3
K79Me3
K9Ac
S10Ph
K56Ac
K18Ac
R3Me3
K20Me3
K5Ac
K16Ac
K8Ac
-600 -400 -200 0 200 400 600 800 1000
-0.5 0 0.5 1 1.5 2 2.5 3 3.5 4

Figure S9

(a)

	H3S10A (108)	H3K56A (108)	H3K18A (108)	H4K20A (108)	H4K5A (108)	H4K16A (108)
H3S10A (108)						
H3K56A (108)	46 (27.1%)					
H3K18A (108)	46 (27.1%)	25 (13.1%)				
H4K20A (108)	56 (35.0%)	53 (32.5%)	24 (12.5%)			
H4K5A (108)	37 (20.7%)	15 (7.5%)	65 (43.0%)	14 (6.9%)		
H4K16A (108)	70 (47.9%)	41 (23.4%)	48 (28.6%)	62 (40.3%)	36 (20.0%)	

(b)

	H3S10A-S (108)	H3K56A-S (108)	H4K5A-S (108)	H4K16A-S (108)	H4K20A-S (108)	H3K18A-S (108)
H3S10A-S (108)						
H3K56A-S (108)	7 (3.3%)					
H4K5A-S (108)	6 (2.9%)	2 (0.9%)				
H4K16A-S (108)	11 (5.4%)	8 (3.8%)	7 (3.3%)			
H4K20A-S (108)	14 (6.9%)	11 (5.4%)	2 (0.9%)	13 (6.4%)		
H3K18A-S (108)	8 (3.8%)	3 (1.4%)	17 (8.5%)	14 (6.9%)	1 (0.5%)	

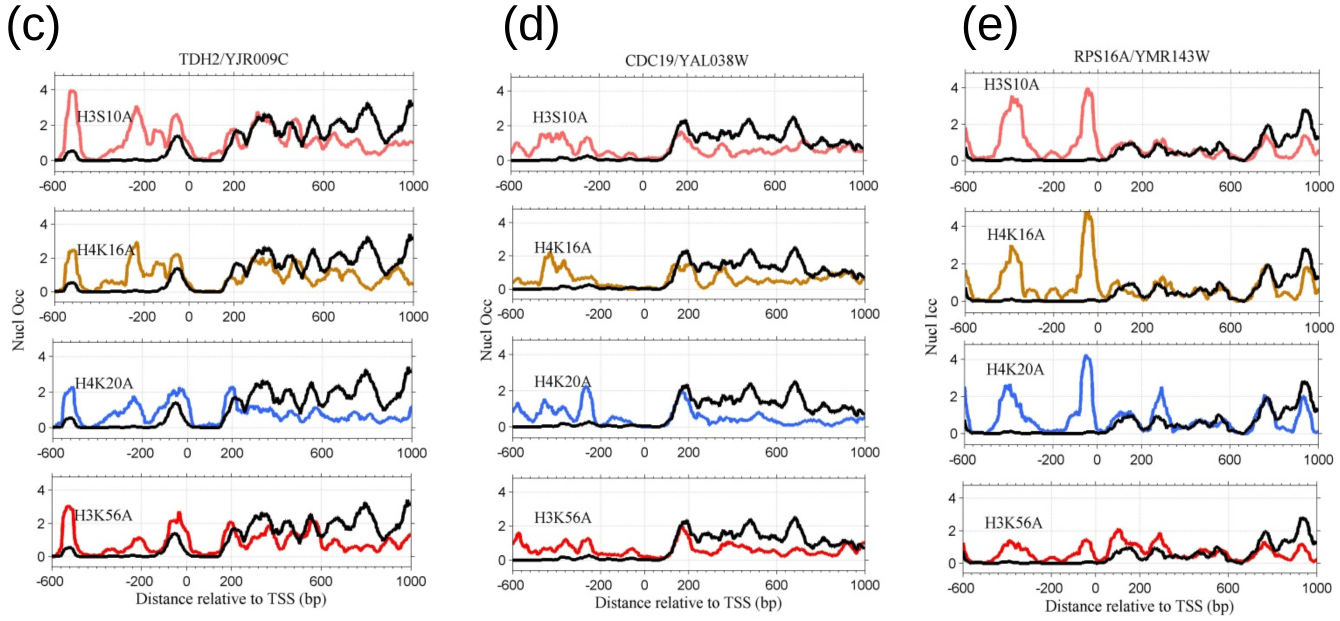

Figure S10

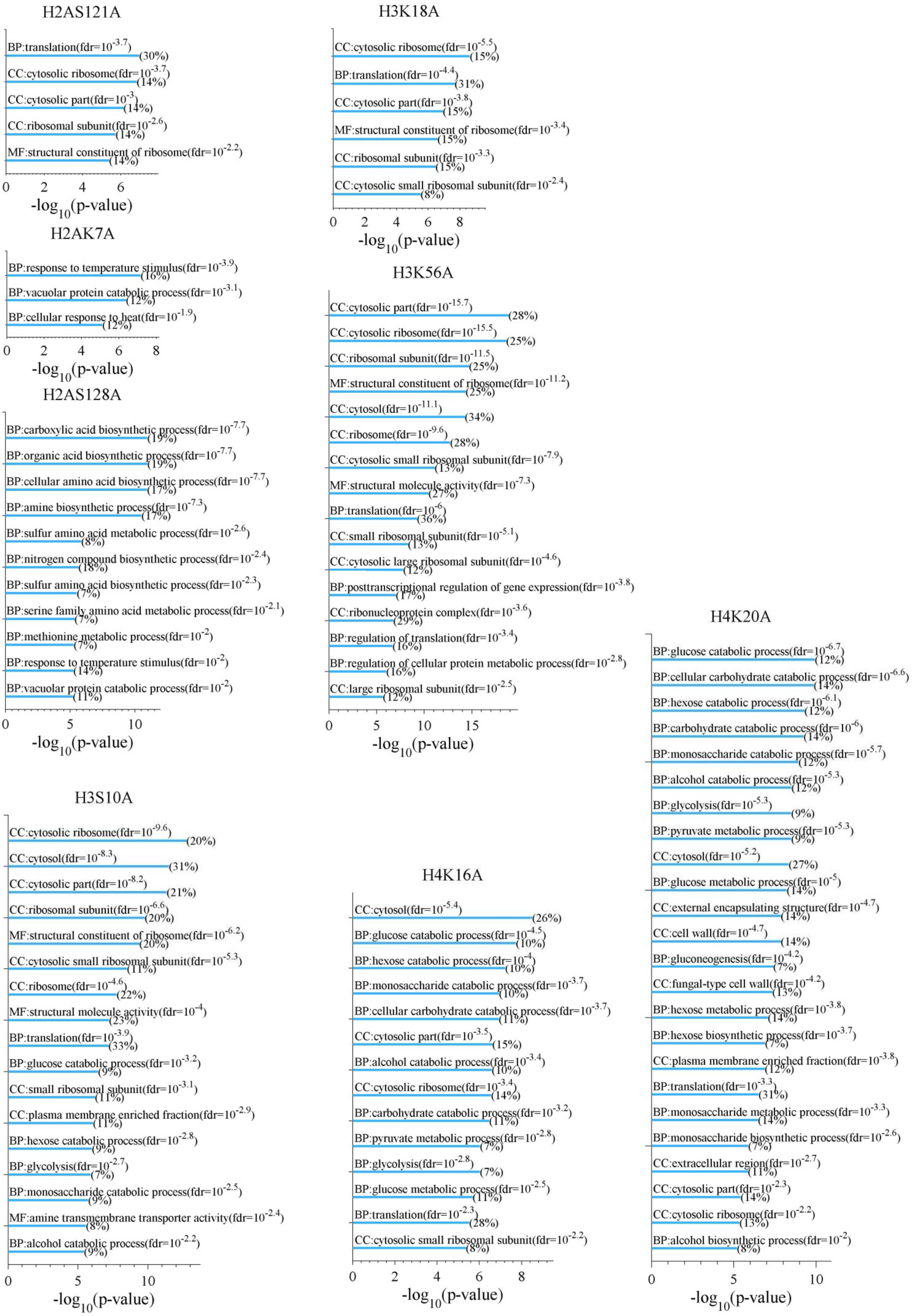
H2AS121A
BP:translation(fdr=$10^{-3.7}$) (30%)
CC:cytosolic ribosome(fdr=$10^{-3.7}$) (14%)
CC:cytosolic part(fdr=10^{-3}) (14%)
CC:ribosomal subunit(fdr=$10^{-2.6}$) (14%)
MF:structural constituent of ribosome(fdr=$10^{-2.2}$) (14%)
0 2 4 6
-log_{10}(p-value)
H2AK7A
BP:response to temperature stimulus(fdr=$10^{-3.9}$) (16%)
BP:vacuolar protein catabolic process(fdr=$10^{-3.1}$) (12%)
BP:cellular response to heat(fdr=$10^{-1.9}$) (12%)
0 2 4 6 8
-log_{10}(p-value)
H2AS128A
BP:carboxylic acid biosynthetic process(fdr=$10^{-7.7}$) (19%)
BP:organic acid biosynthetic process(fdr=$10^{-7.7}$) (19%)
BP:cellular amino acid biosynthetic process(fdr=$10^{-7.7}$) (17%)
BP:amine biosynthetic process(fdr=$10^{-7.3}$) (17%)
BP:sulfur amino acid metabolic process(fdr=$10^{-2.6}$) (8%)
BP:nitrogen compound biosynthetic process(fdr=$10^{-2.4}$) (18%)
BP:sulfur amino acid biosynthetic process(fdr=$10^{-2.3}$) (7%)
BP:serine family amino acid metabolic process(fdr=$10^{-2.1}$) (7%)
BP:methionine metabolic process(fdr=10^{-2}) (7%)
BP:response to temperature stimulus(fdr=10^{-2}) (14%)
BP:vacuolar protein catabolic process(fdr=10^{-2}) (11%)
0 5 10
-log_{10}(p-value)
H3K18A
CC:cytosolic ribosome(fdr=$10^{-5.5}$) (15%)
BP:translation(fdr=$10^{-4.4}$) (31%)
CC:cytosolic part(fdr=$10^{-3.8}$) (15%)
MF:structural constituent of ribosome(fdr=$10^{-3.4}$) (15%)
CC:ribosomal subunit(fdr=$10^{-3.3}$) (15%)
CC:cytosolic small ribosomal subunit(fdr=$10^{-2.4}$) (8%)
0 2 4 6 8
-log_{10}(p-value)
H3K56A
CC:cytosolic part(fdr=$10^{-15.7}$) (28%)
CC:cytosolic ribosome(fdr=$10^{-15.5}$) (25%)
CC:ribosomal subunit(fdr=$10^{-11.5}$) (25%)
MF:structural constituent of ribosome(fdr=$10^{-11.2}$) (25%)
CC:cytosol(fdr=$10^{-11.1}$) (34%)
CC:ribosome(fdr=$10^{-9.6}$) (28%)
CC:cytosolic small ribosomal subunit(fdr=$10^{-7.9}$) (13%)
MF:structural molecule activity(fdr=$10^{-7.3}$) (27%)
BP:translation(fdr=10^{-6}) (36%)
CC:small ribosomal subunit(fdr=$10^{-5.1}$) (13%)
CC:cytosolic large ribosomal subunit(fdr=$10^{-4.6}$) (12%)
BP:posttranscriptional regulation of gene expression(fdr=$10^{-3.8}$) (17%)
CC:ribonucleoprotein complex(fdr=$10^{-3.6}$) (29%)
BP:regulation of translation(fdr=$10^{-3.4}$) (16%)
BP:regulation of cellular protein metabolic process(fdr=$10^{-2.8}$) (16%)
CC:large ribosomal subunit(fdr=$10^{-2.5}$) (12%)
0 5 10 15
-log_{10}(p-value)
H4K20A
BP:glucose catabolic process(fdr=$10^{-6.7}$) (12%)
BP:cellular carbohydrate catabolic process(fdr=$10^{-6.6}$) (14%)
BP:hexose catabolic process(fdr=$10^{-6.1}$) (12%)
BP:carbohydrate catabolic process(fdr=10^{-6}) (14%)
BP:monosaccharide catabolic process(fdr=$10^{-5.7}$) (12%)
BP:alcohol catabolic process(fdr=$10^{-5.3}$) (12%)
BP:glycolysis(fdr=$10^{-5.3}$) (9%)
BP:pyruvate metabolic process(fdr=$10^{-5.3}$) (9%)
CC:cytosol(fdr=$10^{-5.2}$) (27%)
BP:glucose metabolic process(fdr=10^{-5}) (14%)
CC:external encapsulating structure(fdr=$10^{-4.7}$) (14%)
CC:cell wall(fdr=$10^{-4.7}$) (14%)
BP:gluconeogenesis(fdr=$10^{-4.2}$) (7%)
CC:fungal-type cell wall(fdr=$10^{-4.2}$) (13%)
BP:hexose metabolic process(fdr=$10^{-3.8}$) (14%)
BP:hexose biosynthetic process(fdr=$10^{-3.7}$) (7%)
CC:plasma membrane enriched fraction(fdr=$10^{-3.8}$) (12%)
BP:translation(fdr=$10^{-3.3}$) (31%)
BP:monosaccharide metabolic process(fdr=$10^{-3.3}$) (14%)
BP:monosaccharide biosynthetic process(fdr=$10^{-2.6}$) (7%)
CC:extracellular region(fdr=$10^{-2.7}$) (11%)
CC:cytosolic part(fdr=$10^{-2.3}$) (14%)
CC:cytosolic ribosome(fdr=$10^{-2.2}$) (13%)
BP:alcohol biosynthetic process(fdr=10^{-2}) (8%)
0 5 10
-log_{10}(p-value)
H3S10A
CC:cytosolic ribosome(fdr=$10^{-9.6}$) (20%)
CC:cytosol(fdr=$10^{-8.3}$) (31%)
CC:cytosolic part(fdr=$10^{-8.2}$) (21%)
CC:ribosomal subunit(fdr=$10^{-6.6}$) (20%)
MF:structural constituent of ribosome(fdr=$10^{-6.2}$) (20%)
CC:cytosolic small ribosomal subunit(fdr=$10^{-5.3}$) (11%)
CC:ribosome(fdr=$10^{-4.6}$) (22%)
MF:structural molecule activity(fdr=10^{-4}) (23%)
BP:translation(fdr=$10^{-3.9}$) (33%)
BP:glucose catabolic process(fdr=$10^{-3.2}$) (9%)
CC:small ribosomal subunit(fdr=$10^{-3.1}$) (11%)
CC:plasma membrane enriched fraction(fdr=$10^{-2.9}$) (11%)
BP:hexose catabolic process(fdr=$10^{-2.8}$) (9%)
BP:glycolysis(fdr=$10^{-2.7}$) (7%)
BP:monosaccharide catabolic process(fdr=$10^{-2.5}$) (9%)
MF:amine transmembrane transporter activity(fdr=$10^{-2.4}$) (8%)
BP:alcohol catabolic process(fdr=$10^{-2.2}$) (9%)
0 5 10
-log_{10}(p-value)
H4K16A
CC:cytosol(fdr=$10^{-5.4}$) (26%)
BP:glucose catabolic process(fdr=$10^{-4.5}$) (10%)
BP:hexose catabolic process(fdr=10^{-4}) (10%)
BP:monosaccharide catabolic process(fdr=$10^{-3.7}$) (10%)
BP:cellular carbohydrate catabolic process(fdr=$10^{-3.7}$) (11%)
CC:cytosolic part(fdr=$10^{-3.5}$) (15%)
BP:alcohol catabolic process(fdr=$10^{-3.4}$) (10%)
CC:cytosolic ribosome(fdr=$10^{-3.4}$) (14%)
BP:carbohydrate catabolic process(fdr=$10^{-3.2}$) (11%)
BP:pyruvate metabolic process(fdr=$10^{-2.8}$) (7%)
BP:glycolysis(fdr=$10^{-2.8}$) (7%)
BP:glucose metabolic process(fdr=$10^{-2.5}$) (11%)
BP:translation(fdr=$10^{-2.3}$) (28%)
CC:cytosolic small ribosomal subunit(fdr=$10^{-2.2}$) (8%)
0 2 4 6 8
-log_{10}(p-value)

Figure S11

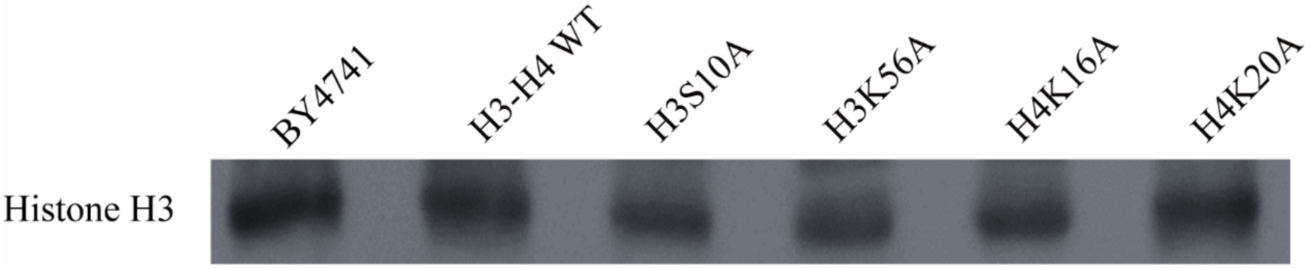

Figure S12

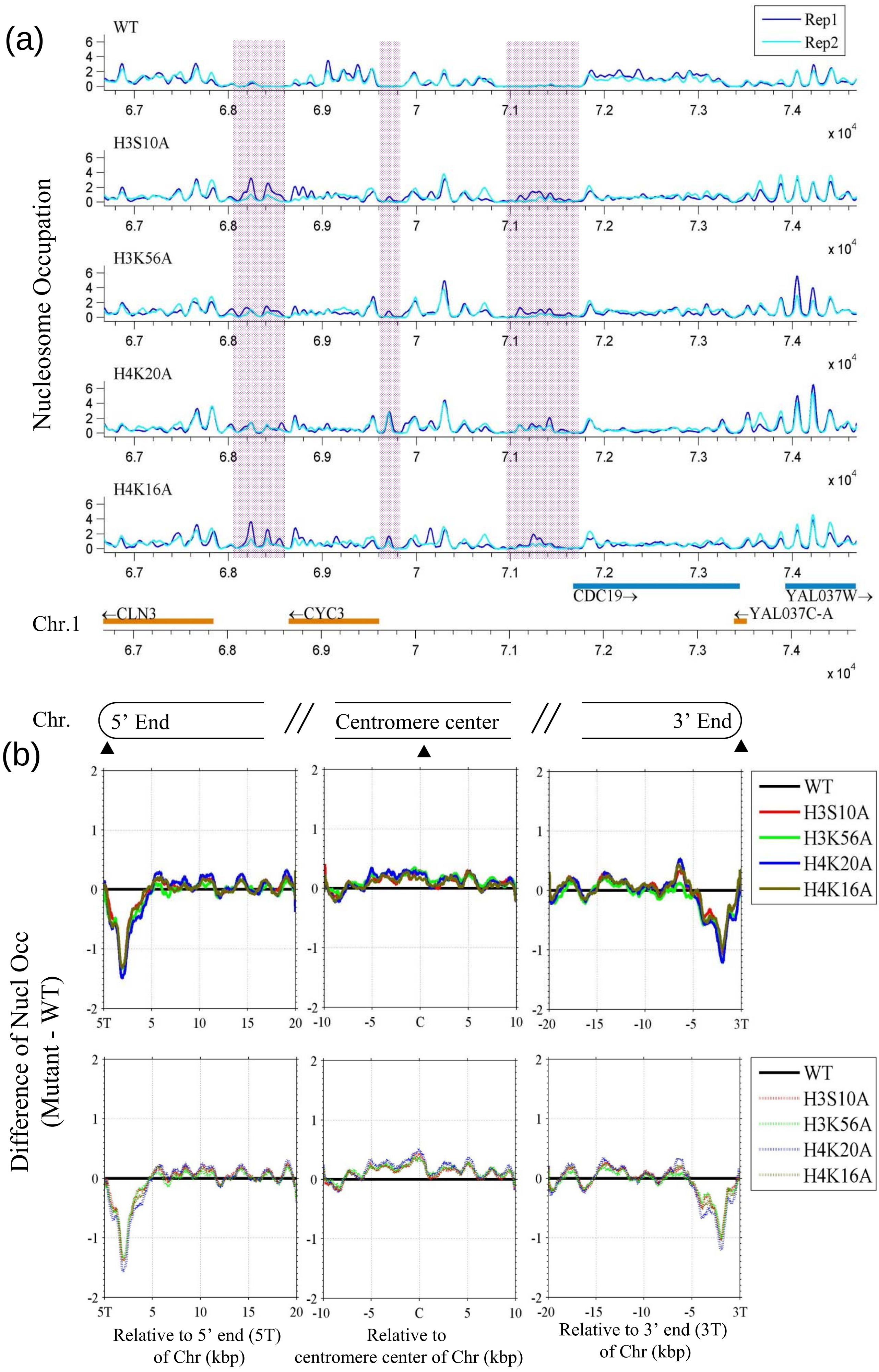
(a)
WT
Rep1
Rep2
H3S10A
H3K56A
H4K20A
H4K16A
Nucleosome Occupation
x 10^4
CDC19→
YAL037W→
←CLN3
←CYC3
←YAL037C-A
Chr.1
Chr.
5' End
Centromere center
3' End
(b)
WT
H3S10A
H3K56A
H4K20A
H4K16A
Difference of Nucl Occ
(Mutant - WT)
Relative to 5' end (5T) of Chr (kbp)
Relative to centromere center of Chr (kbp)
Relative to 3' end (3T) of Chr (kbp)
